# Intranasal booster drives class switching and homing of memory B cells for mucosal IgA response

**DOI:** 10.1172/jci.insight.198045

**Published:** 2025-12-23

**Authors:** Si Chen, Zhengyuan Zhang, Zihan Lin, Li Yin, Lishan Ning, Wenming Liu, Qian Wang, Chenchen Yang, Bo Feng, Ying Feng, Yongping Wang, Hengchun Li, Ping He, Huan Liang, Yichu Liu, Zhixia Li, Bo Liu, Yang Li, Diana Boraschi, Linbing Qu, Xuefeng Niu, Nanshan Zhong, Pingchao Li, Ling Chen

**Affiliations:** 1 State Key Laboratory of Respiratory Disease, National Clinical Research Center for Respiratory Disease, the First Affiliated Hospital of Guangzhou Medical University, Guangzhou, China.; 2 Guangzhou Institute of Infectious Disease, Guangzhou Eighth People’s Hospital, Guangzhou Medical University, Guangzhou, China.; 3 State Key Laboratory of Respiratory Disease, Human Cell Lineage Atlas Facility, Guangdong Provincial Key Laboratory of Biocomputing, Institute of Drug Discovery, Guangzhou Institutes of Biomedicine and Health, Chinese Academy of Sciences, Guangzhou, China.; 4 Guangzhou National Laboratory, Guangzhou, China.; 5 University of Chinese Academy of Sciences, Beijing, China.; 6 Shenzhen Institutes of Advanced Technology, Shenzhen, China.

**Keywords:** Immunology, Infectious disease, Public Health, B cells, Immunoglobulins, Vaccines

## Abstract

Mucosal secretory IgA (sIgA) plays a central role in protecting against the invasion of respiratory pathogen via the upper respiratory tract. To understand how intranasal booster induces mucosal sIgA response in humans, we first used liquid chromatography–tandem mass spectrometry for peptide identification of immunoglobulin (MS Ig-seq) and single-cell B cell receptor sequencing (scBCR-seq) to identify 42 mucosal spike-specific sIgA monoclonal antibodies (mAbs) after intranasal booster. These mucosal sIgA mAbs exhibited enhanced neutralization up to 100-fold against SARS-CoV-2 variants compared with their monomeric IgG and IgA isotypes. Deep sequencing and longitudinal analysis of B cell receptor repertoires revealed that intranasal booster restimulates memory B cells primed by intramuscular vaccination to undergo IgA class switching, somatic hypermutation, and clonal expansion. Single-cell RNA-seq (scRNA-seq) revealed that intranasal booster upregulated the expression of mucosal homing receptors in spike-specific IgA-expressing B cells. This increase coincided with a transient increase of cytokines and chemokines that facilitate B cell recruitment in the nasal mucosa. Our findings demonstrate that intranasal booster can be an effective strategy for inducing upper respiratory mucosal sIgA and establishing mucosal immune protection.

## Abstract 2

Intranasal booser elicits mucosal IgA response by promoting class switching and mucosal homing of primed memory B cells

## Graphical abstract

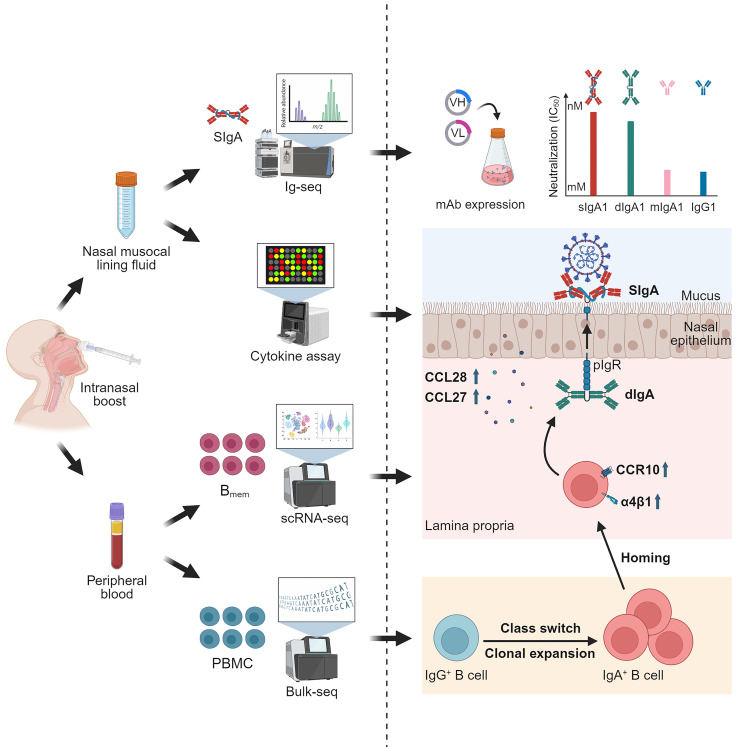

## Introduction

The upper airway mucosa serves as the frontline defense against respiratory pathogens, with secretory IgA (sIgA) playing a central role in mucosal immune protection. During the SARS-CoV-2 pandemic, individuals who received intramuscularly injected vaccines and experienced a natural infection were better protected against SARS-CoV-2 Omicron infection than individuals who received only injected vaccines (1). This is because injected vaccines cannot induce sIgA response on the upper respiratory mucosa, which is the entry point for SARS-CoV-2 infection (2, 3). Higher levels of spike-specific sIgA in nasal mucosal fluids or saliva correlate with greater protection against Omicron breakthrough infections (4, 5). As the predominant immunoglobulin on mucosal surfaces, sIgA is mainly present as a dimeric (dIgA) and polymeric forms composed of 2 or more IgA monomers linked by a joining (J) chain (6). sIgA is produced locally by IgA^+^ B cells as dIgA and transported through the polymeric immunoglobulin receptor (pIgR) at the basolateral surfaces of mucosal epithelial cells; it is then released into the lumen as sIgA. Because of its transport mechanism, sIgA can also neutralize pathogens within mucosal epithelial cells. sIgA provides local mucosal immunity by neutralizing pathogens and preventing their adhesion to epithelial cells (7–9).

Intramuscularly injected SARS-CoV-2 vaccines can decrease the severity of disease and mortality but fail to effectively prevent the infection and transmission due to their inability to elicit mucosal sIgA on the upper respiratory tract, especially the nasal passage (2, 10). We previously reported a clinical study using 2-dose intranasal booster with Ad5-S-Omicron (NB2155), which demonstrated a 52-fold geometric mean fold increase (GMFI) of nasal mucosal spike-specific sIgA against 10 SARS-CoV-2 variants at 14 days after vaccination. In the meantime, the serum geometric mean titers (GMTs) against WT, BA.1, and BA.5 increased from 53, 13, and 17 before vaccination to 1,158, 688, and 303 after the 2-dose regimen. This was verified by a cytopathic plaque-forming assay, which showed that the GMT increased from 1:10 to 1:128 and 1:77 against authentic BA.1 and BA.5 viruses (11). Intriguingly, all individuals who received 2 doses of the vaccine for at least 3 days remained uninfected during the Omicron wave between December 2022 and January 2023 (11). Therefore, the high level of spike-specific sIgA on the upper respiratory mucosa is crucial to completely blocking the invading SARS-CoV-2.

Vaccination at the mucosal surfaces is considered a highly effective strategy for inducing robust protective immunity in the respiratory tract, gastrointestinal tract, and other mucosal sites (12–14). Studies in mouse models have shown that the expression of homing markers induced by mucosal vaccination or infection can direct the migration of activated antigen-specific B cells to local or distal mucosal sites (15, 16). The nasal tract harbors the nasal-associated lymphoid tissue (NALT), which is part of the mucosa-associated lymphoid tissue (MALT) and plays an essential role in B cell maturation into plasma cells that produce mucosal sIgA (17, 18). However, the molecular and functional properties of sIgA antibodies elicited in human nasal mucosa remain poorly understood. A notable gap in the field of human mucosal immunity is that few studies have addressed the isolation and characterization of mAbs from mucosal sites in humans. While spike-specific IgG mAbs have been cloned from human peripheral blood and converted to sIgA (19), the lack of nasal mucosa-associated sIgA mAbs hinders our ability to investigate antigen specificity and neutralizing capacity of mucosal antibody response to nasal vaccines or natural infection. The induction, class switching, and homing of IgA-expressing B cells to the human nasal mucosa require further investigation. In this study, firstly, we attempted to clone mucosal antigen-specific sIgA mAbs following intranasal booster vaccination. Next, we analyzed the presence and evolution of these mucosal mAbs and their B cell clonotypes in B cell receptor (BCR) repertoires following either injected vaccines or intranasal boosters. Along with the data from scRNA-seq of memory B cells, we sought to understand the stimulation and expansion of spike-specific memory B cells, class switching to IgA, and the homing of IgA^+^ B cells to the nasal mucosa.

## Results

### Intranasal booster-induced mucosal sIgA could be up to 100-fold more potent than serum IgG and IgA in neutralizing SARS-CoV-2 variants.

**Figure 1:**
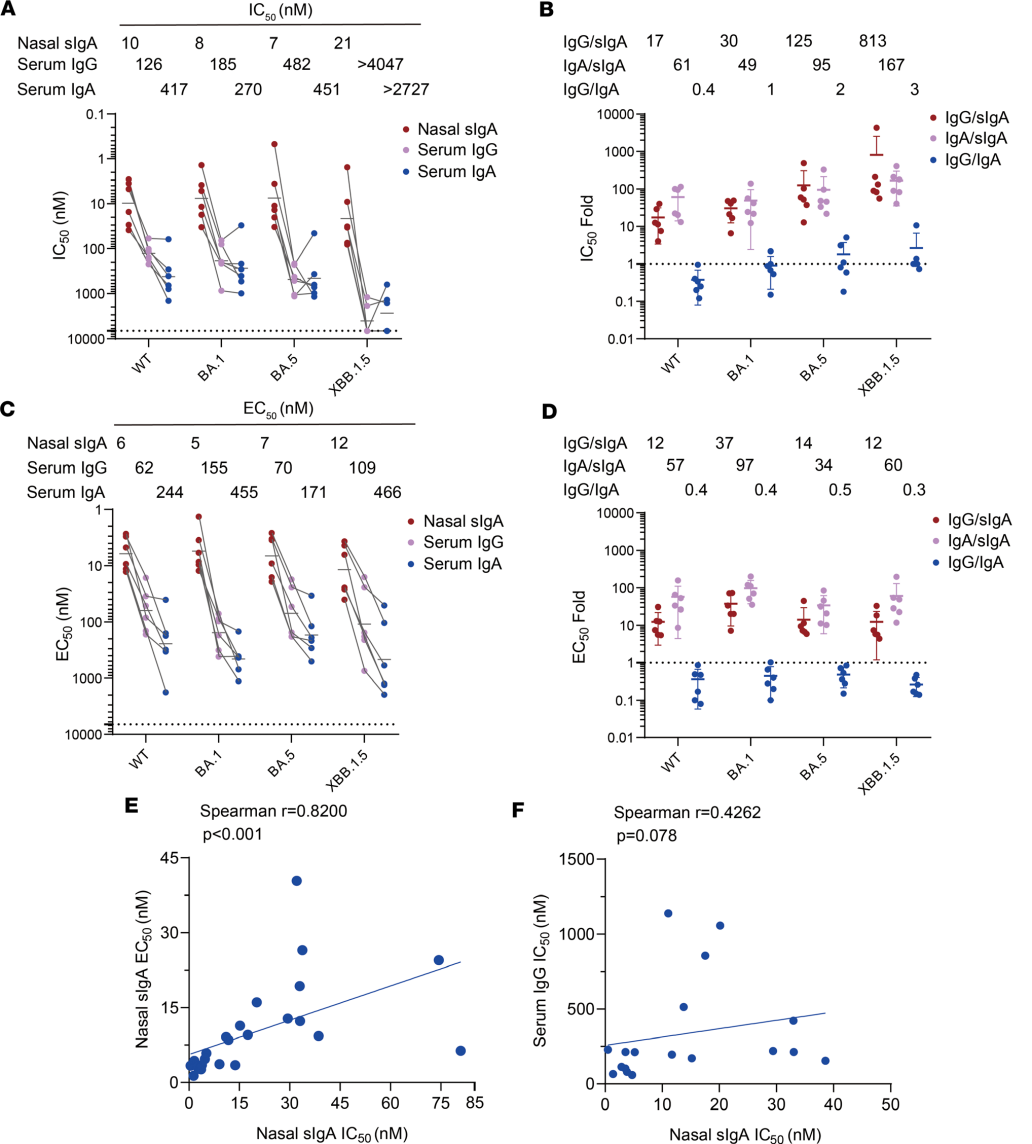
Neutralizing and spike-binding activities of purified nasal sIgA, serum IgG, and IgA. (**A**) Neutralizing activities of 6 paired purified nasal sIgA, serum IgG, and serum IgA samples against WT, BA.1, BA.5, and XBB.1.5 were assessed using a lentivirus-based pseudovirus system. The results are presented as 50% inhibitory concentration (IC_50_) in nM. Samples showing no detectable neutralization activity at the highest concentration (1,000 μg/mL) were assigned an IC_50_ value of 6,666.7 nM (dashed line), representing no neutralization. Geomean values (*n* = 6) are reported above the graph. (**B**) The ratios of IC_50_ between paired IgG/sIgA, IgA/sIgA, and IgG/IgA for each donor are presented. Mean values (*n* = 6) are reported above the graph. (**C**) Binding activities of 6 paired purified nasal sIgA, serum IgG and serum IgA samples against WT, BA.1, BA.5, and XBB.1.5 spike proteins were measured by ELISA. Results are presented as 50% effective concentration (EC_50_) in nM. Data are calculated as geomean (*n* = 6) and reported above the graph. (**D**) The ratios of EC_50_ between paired IgG/sIgA, IgA/sIgA, and IgG/IgA for each donor are presented. Mean values (*n* = 6) are reported above the graph. (**E** and **F**) Correlation analysis was performed between: nasal sIgA neutralization activity (IC_50_) and binding activity (EC_50_) (**E**), nasal sIgA and serum IgG neutralization activity (IC_50_) (**F**). Statistical significance was determined using Spearman’s rank correlation (coefficients and *P* values are shown).

All donors previously received 2 injections of inactivated whole-virus vaccine more than 6 months ago and have never been infected with SARS-CoV-2 (Supplemental Table 1; supplemental material available online with this article; https://doi.org/10.1172/jci.insight.198045DS1). There were no substantial nasal spike-specific IgA before intranasal vaccination. Nasal lavages and serum samples from each donor were collected within 3–6 weeks after 2 doses of intranasal spray with Ad5-Omicron spike vaccine, an adenovirus type 5 carrying Omicron BA.1 spike (Supplemental Table 1). Paired nasal sIgA, serum IgG, and serum IgA from the same individuals were purified using affinity chromatography. The average yield of purified nasal sIgA from the nasal lavages of the 6 donors was 1.36 ± 0.31 μg/mL, while the average yields from serum were 9.13 ± 2.38 mg/mL for IgG and 0.75 ± 0.15 mg/mL for IgA (Supplemental Table 7). SDS-PAGE and Western blot analysis confirmed that nasal sIgA from all donors are dimeric and are polymeric forms with molecular weight 400 kD or higher (Supplemental Figure 1). In contrast, serum IgG and IgA are monomeric with molecular weight 150 kD and 160 kD, respectively (Supplemental Figure 1). Nasal sIgA was compared with the same person’s serum IgG and IgA for their neutralizing and spike binding activities. On average, nasal sIgA was 17-, 30-, 125-, and 813-fold more potent than serum IgG in neutralizing SARS-CoV-2 WT, BA.1, BA.5, and XBB.1.5 pseudoviruses. Similarly, nasal sIgA was 61, 49, 95, and 167-fold more potent than serum IgA in neutralizing WT, BA.1, BA.5, and XBB.1.5 (Figure 1, A and B, and Supplemental Tables 2 and 3). For each donor, nasal sIgA was 12- to 37-fold stronger than serum IgG and was 34- to 97-fold stronger than serum IgA in binding to spike proteins of WT, BA.1, BA.5, and XBB.1.5, respectively (Figure 1, C and D, Supplemental Tables 2 and 3). We also accessed the percentage of spike-specific antibodies in the purified sIgA, serum IgA, and serum IgG using mAb 719-1, a broadly neutralizing mAbs (this study) in its respective isotypes as a reference standard. Among these paired nasal lavage and serum samples, there was an average of 0.83% (0.11%–2.38%), 0.59% (0.08%–1.34%), 0.25% (0.03%–0.53%), and 0.20% (0.02%–0.52%) of WT, BA.1, BA.5, and XBB.1.5 spike-specific sIgA in total nasal sIgA. There was an average of 0.33% (0.13%–0.68%), 0.37% (0.15%–0.74%), 0.17% (0.11%–0.34%), and 0.13% (0.07%–0.19%) of WT, BA.1, BA.5, and XBB.1.5 spike-specific IgG in total serum IgG. There was an average of 0.13% (0.02%–0.28%), 0.16% (0.02%–0.40%), 0.05% (0.01%–0.14%), and 0.04% (0.01%–0.07%) of WT, BA.1, BA.5, and XBB.1.5 spike-specific IgA in total serum IgA (Supplemental Figure 5A). We then compared the neutralizing activity of nasal sIgA, serum IgA, and serum IgG based on equal amounts of spike-specific antibodies for each paired antibody isotypes. On average, nasal sIgA exhibited 10-, 23-, 85-, and 2,162-fold greater potency than serum IgG in neutralizing SARS-CoV-2 WT, BA.1, BA.5, and XBB.1.5 pseudoviruses, respectively. Nasal sIgA exhibited 10-, 10-, 17-, and 720-fold more potent than serum IgA in neutralizing WT, BA.1, BA.5, and XBB.1.5 pseudoviruses, respectively (Supplemental Figure 5, B and C). Spearman correlation analysis showed that nasal sIgA neutralizing activity was highly correlated with sIgA spike binding activity (r = 0.8200, *P* < 0.001), but not with serum IgG neutralizing activity (r = 0.4262, *P* = 0.078) (Figure 1, E and F). Therefore, nasal sIgA is much more potent than serum IgG and IgA in neutralizing and binding activities. The disparity between nasal sIgA and serum IgG or serum IgA was even more dramatic for lately emerged Omicron XBB.1.5, demonstrating nasal sIgA has enhanced broadly neutralizing breadth than serum antibodies in neutralizing all early and late SARS-CoV-2 variants that we tested.

### Obtaining mucosa–associated sIgA mAbs using a combination of mass spectrometry and B cell sequencing.

**Figure 2:**
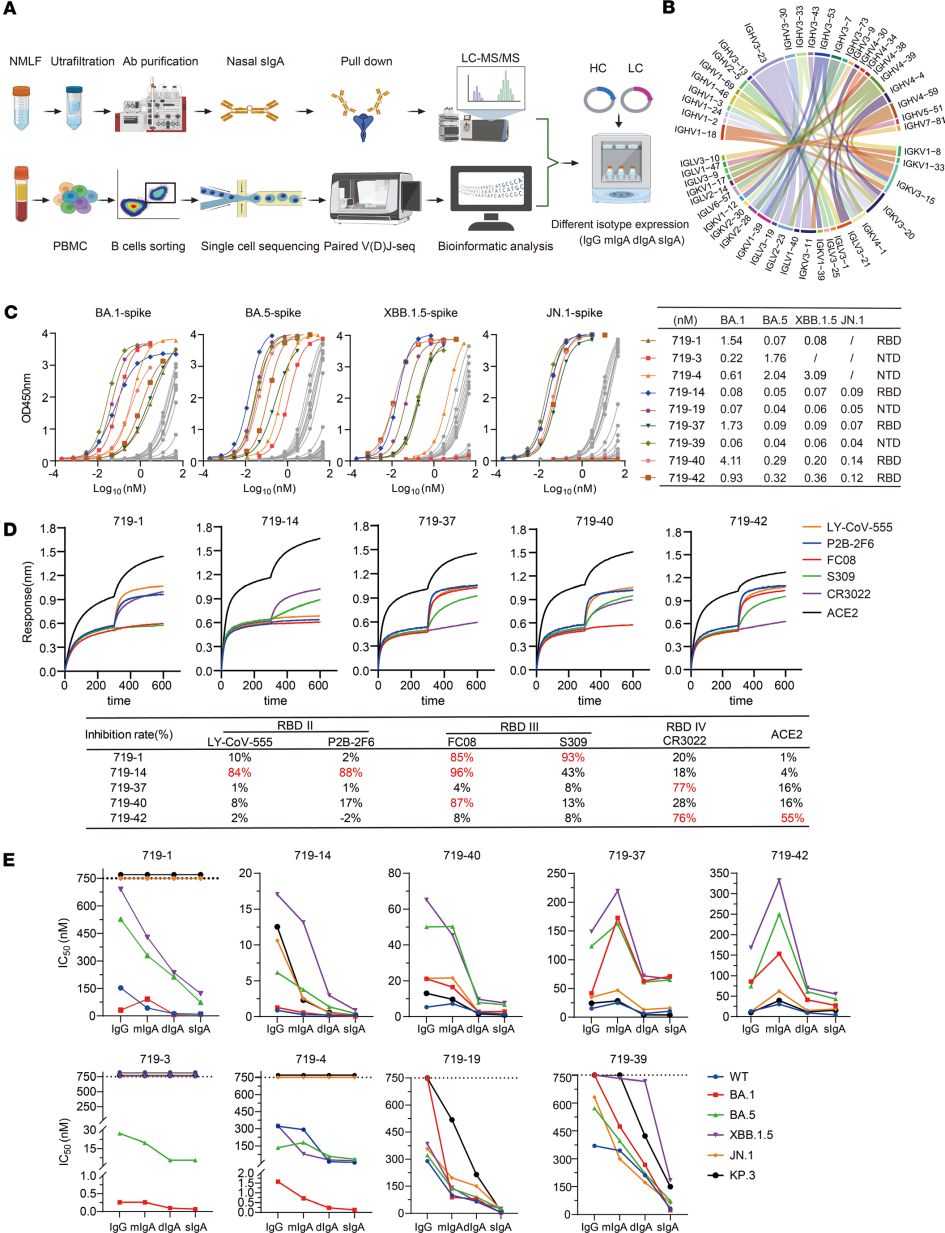
Identification of mucosal sIgA mAbs using MS Ig-seq and scBCR-seq. (**A**) Scheme of the study. The schematic was drawn using BioRender. For MS Ig-seq, BA.1 spike-specific sIgA from a donor was isolated using magnetic beads coupled with BA.1 spike, digested with trypsin, and analyzed using LC-MS/MS. A personalized antibody repertoire was constructed by scBCR-seq from the same donor. cDNAs encoding the heavy chain and light chain of respective antibodies were synthesized and cloned into expression plasmids. (**B**) The germline heavy- and light-chain gene usage (IGHV, IGKV, and IGLV) and their pairing combinations among the 45 isolated antibodies were visualized using a chord diagram. Each arc represents a heavy-chain and light-chain germline gene pair, the width of which is proportional to the corresponding antibody count. (**C**) Binding of mucosal mAbs (719-1 to 45, IgG) to variant spike proteins (BA.1, BA.5, XBB.1.5, and JN.1) was measured by ELISA. The chart shows mAbs with EC50 below 5 nM against at least 2 variants. (**D**) BLI-based competition assays were performed between RBD-binding mAbs 719-1, 719-14, 719-37, 719-40, 719-42, and known antibodies targeting distinct RBD epitopes (LY-CoV-555, P2B-2F6, FC08, S309 and CR3022) or ACE2. The competition percent between each isolated mAb and the known antibodies or ACE2 is presented in the table. (**E**) Assessment of the neutralizing potency of mAbs 719-1, 719-3, 719-4, 719-14, 719-19, 719-37, 719-39, 719-40, and 719-42 expressed in IgG, mIgA, dIgA, and sIgA forms against pseudoviruses expressing spike from WT and Omicron subvariants BA.1, BA.5, XBB.1.5, JN.1, and KP.3. Data are shown as IC_50_.

Since few studies have focused on the isolation and characterization of mAbs from nasal mucosa in humans, we combined liquid chromatography–tandem mass spectrometry (LC-MS/MS) for peptide identification of spike-binding sIgA (MS Ig-seq) with single-cell BCR sequencing (scBCR-seq) to obtain paired heavy- and light-chain sequences, which were then cloned into expression plasmids for recombinant production of mucosa-associated sIgA mAbs (Figure 2A). Spike-specific sIgA was purified from the nasal lavage of a donor (Donor 1) at 4 weeks after the second intranasal booster and subsequently analyzed by LC-MS/MS. Peptide identification from the raw LC-MS/MS data was performed using Proteome Discoverer and PEAKS Studio software, which requires a comprehensive antibody database for the search. Accordingly, we constructed a personalized antibody repertoire by performing scBCR-seq on peripheral blood mononuclear cell–derived (PBMC-derived) B cells from the same donor after each vaccination (Supplemental Table 4). A total of 33,675 BCR sequences and 13,433 peptide sequences were obtained (Supplemental Table 4). Alignment of sIgA peptide sequences with BCR immunoglobulin heavy chain (IgH) sequences led to the identification of 45 heavy-chain IgH sequences and their corresponding immunoglobulin light chain (IgL) sequences (Figure 2B and Supplemental Figure 2B). These 45 mucosal mAbs are contributed by 24 IgH germline genes, including IGHV3-23 (7 of 45, 15.6%), IGHV4-39 (4 of 45, 8.9%), IGHV1-18, IGHV3-53, and IGHV4-59 (3 of 45, 6.7%), and by 23 IgL germline genes, including IGKV3-15 (6 of 45, 13.3%), IGKV3-20 (5 of 45, 11.1%), IGKV1-39, IGKV3-11, IGKV4-1, IGLV2-23, and IGLV3-21 (3 of 45, 6.7%) (Figure 2B). The cDNAs were then synthesized and cloned into expression plasmids for production of mAbs in different isotypes. Of the 42 mAbs that were successfully expressed and purified in IgG1 form, 30 mAbs showed strong binding activity to the BA.1 and BA.5 spike proteins, while 12 mAbs showed weak binding activity. Through ELISA, we found that the successfully expressed mAbs bind to either the receptor-binding domain (RBD) or the N-terminal domain (NTD) (Figure 2C and Supplemental Table 5).

### Nasal dIgA and sIgA mAbs have more potent neutralizing and spike-binding activities than their monomeric IgG and IgA isoforms.

Nine mAbs with strong neutralizing and spike-binding activities against BA.5 were selected for further study (Figure 2C and Table 1). mAbs 719-3, 719-4, 719-19, and 719-39 bind to the NTD, whereas 719-1, 719-14, 719-37, 719-40, and 719-42 bind to RBD. mAbs 719-14, 719-19, 719-37, 719-39, 719-40, and 719-42 bind to spike proteins of BA.1, BA.5, XBB.1.5, and JN.1. 719-1 and 719-4 bind to spike proteins of BA.1, BA.5, and XBB.1.5. mAb 719-3 binds to spike proteins of BA.1 and BA.5 (Figure 2C). Using biolayer interferometry (BLI) analysis, we found that 719-1, 719-14, and 719-40 competed for RBD binding with well-known RBD III type mAbs FC08 or S309 (20, 21). mAb 719-14 also competes with LY-CoV-555 and P2B-2F6, 2 well-known RBD II type mAbs (22, 23). mAbs 719-37 and 719-42 compete with CR3022, a well-known RBD IV type mAb (24). mAb 719-42 also showed competition with angiotensin-converting enzyme 2 (ACE2) for binding to spike, suggesting its epitope overlaps with ACE2 binding site (Figure 2D). Therefore, nasal mucosal neutralizing mAbs can target a diverse range of epitopes on spike protein.

We next expressed and purified each of these neutralizing mAbs in 4 isotypes — IgG1, mIgA1, dIgA1, and sIgA1 — and confirmed their molecular weights and assessed purities using SDS-PAGE and Western blot analysis (Supplemental Figure 2A). All sIgA isotypes exhibited greater neutralizing potency than the corresponding IgG or mIgA isotypes against at least 1 SARS-CoV-2 variant, with an average enhancement of 13-fold (Figure 2E and Table 1 and Table 2). Some mAbs in sIgA isotype, such as 719-14 and 719-40, exhibited IC_50_ in subpicomolar range against lately emerged Omicron JN.1 and KP.3. 719-14 sIgA was 58-fold more potent, and 719-40 sIgA was 15-fold more potent than their IgG isotypes in neutralizing KP.3. Interestingly, 719-19 and 719-39 IgG isotypes showed no neutralizing activities against BA.1 and KP.3 (IC_50_ > 750 nM), but their sIgA isotypes showed an IC_50_ of 3.42 nM and 23.63 nM against BA.1, and 41.57 nM and 149.45 nM against KP.3, respectively (Figure 2E and Tables 1 and 2). Some sIgA, such as 719-19 sIgA, exhibited 23-fold enhanced neutralizing potency than dIgA against BA.1, demonstrating secretory component (SC) may have marked enhancement on some sIgA mAbs. Further investigation is required to understand how the addition of SC enhances the neutralizing potency of dIgA. Most mAbs in IgG isotype showed similar neutralizing potency as their mIgA isotypes (Figure 2E and Table 1). We also assessed these mAbs in sIgA, dIgA, mIgA, and IgG isotypes for binding activities with spike proteins of WT, BA.1, BA.5, XBB.1.5, JN.1, and KP.3. Most mAbs in sIgA isotype were 2- to 11-fold more potent than their mIgA and IgG isotypes in binding to at least 1 spike protein (Supplemental Figure 3 and Table 3).

### Intranasal booster drives IgA class switching and clonal expansion of spike-specific memory B cells primed by intramuscular vaccination.

**Figure 3:**
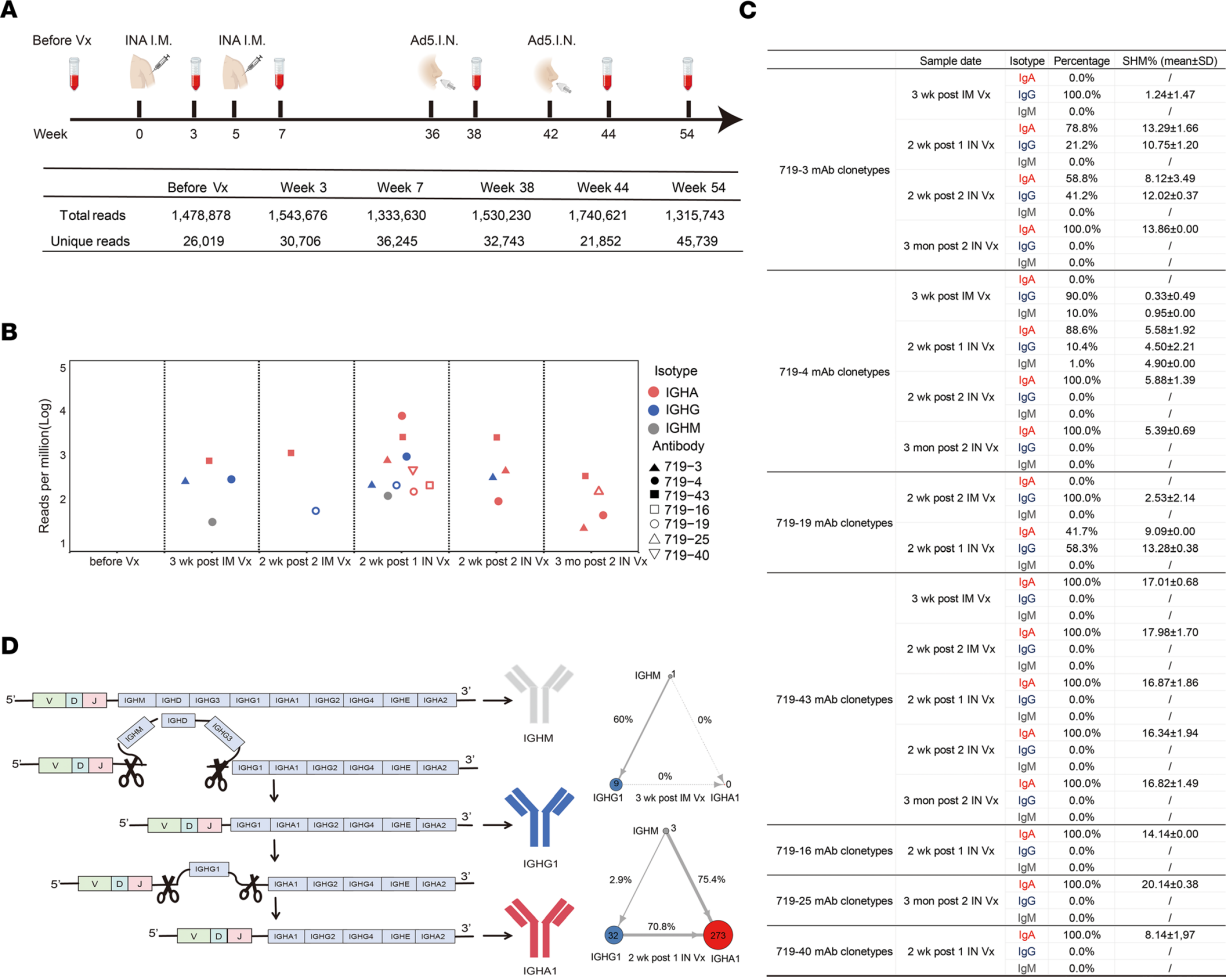
Clonotypes of nasal mucosal mAbs in BCR repertoires at different time points after intramuscular and intranasal vaccination. (**A**) Sample collection scheme for sequencing BCR IgH repertoires. The schematic was drawn using BioRender. Five million peripheral mononuclear cells (PBMC) were obtained from a donor at each time point and subjected to bulk BCR sequencing as described in the methods. These time points are: before vaccination (before Vx), 3 weeks after intramuscular injection with inactivated whole-virus vaccine, 2 weeks after second intramuscular injection with inactivated whole-virus vaccine, 2 weeks after the first intranasal vaccination with Ad5-spike-BA.1, 2 weeks after the second intranasal booster, and 3 months after second intranasal booster. The total number of reads and unique reads in the heavy-chain repertoires are shown in the table. (**B**) The presence of 719-3, 719-4, 719-16, 719-19, 719-25, 719-40, and 719-43 clonotypes in circulating B cells at different time points after vaccination. Different shapes represent distinct clonotypes. (**C**) The isotype percentages and somatic hypermutation rates of 719-3, 719-4, 719-16, 719-19, 719-25, 719-40, and 719-43 clonotypes in circulating B cells at different time points after vaccination. (**D**) Class switch recombination (CSR) patterns of 719-4 clonotypes after intramuscular vaccination and intranasal vaccination. The color of each circle represents an Ig isotype, and the size of the circle reflects the relative number of unique Ig sequences. The arrow represents the direction of class-switching. The arrow’s thickness represents the probability of CSR, and the dashed line indicates no CSR occurrence.

We used deep sequencing to obtain BCR repertoires from PBMCs collected from the same donor before and after each vaccination, for a total of 6 time points. The BCR repertoires at each time point contained between 1.32 and 1.74 million IgH sequences. After quality control processing, an average of 30,000 unique IgH sequences were obtained per time point (Figure 3A). Next, we searched for antibodies that expressed the same clonotypes as those mucosal-derived mAbs. These clonotypes contained the same V and J genes, as well as at least 75% HCDR3 sequence identity. Before any vaccination, no clonotypes of these mAbs could be found in the BCR repertoire. After the first or second intramuscular vaccination, a low number of 719-3, 719-4, and 719-19 clonotypes were found in IgM and IgG isotypes but not in IgA isotype (Figure 3, B and C). Notably, 2 weeks after the first intranasal booster, a substantial number of 719-3, 719-4, and 719-19 in IgA clonotypes appeared, indicating marked IgA class-switching. These IgA clonotypes had higher rates of somatic hypermutation (SHM) than the IgG isotype (Figure 3C). Three months after the intranasal booster, a low number of 719-3 and 719-4 clonotypes in the IgA isotype, but not the IgG isotype, were found (Figure 3, B and C). These results suggest that the spike-specific memory B cells generated by intramuscular priming underwent IgA class-switching and clonal expansion following intranasal booster. The 719-43 clonotype was only found in the IgA isotype after intramuscular vaccination, and it increased after the intranasal booster, suggesting that IgA^+^ memory B cells may also undergo clonal expansion after intranasal booster (Figure 3B). The 719-43 IgA clonotype exhibited a relatively high SHM rate of 17% ± 1.5% (Figure 3C). The clonotypes 719-16, 719-25, and 719-40 could only be found as IgA after the intranasal booster. This suggests that the number of IgA^+^ B cells may have been increased, while the number of IgG^+^ B cells may have been low or largely converted to IgA^+^ B cells (Figure 3, B and C). The 719-4 clonotypes were the most abundant and had all 3 isotypes (IgM, IgG, and IgA) present in their BCR repertoires. This enabled us to calculate the frequency of class-switch recombination (CSR) events (Figure 3D). After intramuscular priming, the probability of CSR from IgM to IgG1 was 60%, and no CSR from IgM to IgA1 or IgG1 to IgA1 was observed. In contrast, after the intranasal booster, the probability of CSR from IgM to IgA1 increased to 75.4%, and the probability of CSR from IgG1 to IgA1 increased to 70.8% (Figure 3D).

We constructed a genealogical tree for clonotypes 719-3 and 719-4, as they were identified in the BCR repertoires after intramuscular vaccination and intranasal booster. The 719-3 clonotype is derived from the IGHV4-59 germline, and its B cells were generated following intramuscular vaccination at a low SHM rate of approximately 1.3%. Following the intranasal booster, the 719-3 clonotype underwent clonal expansion and IgA class-switching, and SHM rate increased to 16.7% (Supplemental Figure 4A). The 719-4 clonotype is derived from the IGHV5-51 germline, and its B cells were generated after intramuscular vaccination with an SHM rate of 1%. After the intranasal booster, the 719-4 clonotype underwent clonal expansion and IgA class switching, and SHM rate increased to 5.9% (Supplemental Figure 4B).

### Spike-specific IgA^+^ B cells express upregulated mucosal homing receptors after intranasal booster.

**Figure 4:**
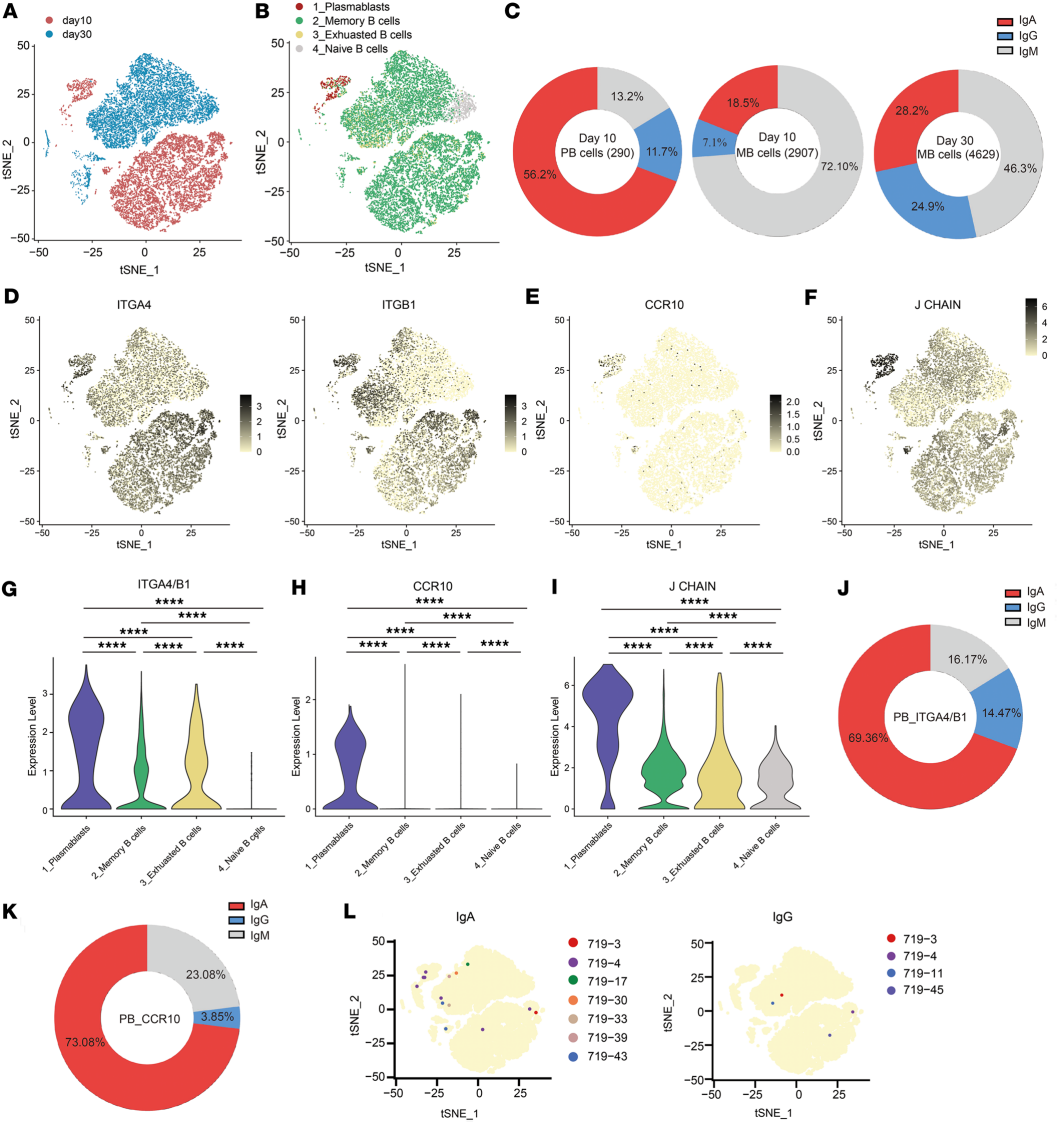
ScRNA-seq of circulating B cells on day 10 and day 30 after intranasal booster. (**A** and **B**) The t-SNE plot shows the cell clustering of 16,923 immune cells at 2 time points (**A**) and specific B cell types (**B**). The time points of cell clustering are separated according to color, with red representing day 10 and blue representing day 30 (**A**). Red represents plasmablasts, green represents memory B cells, gray represents naive B cells, and yellow represents exhausted B cells (**B**). (**C**) Pie charts depicting the relative proportions of IgA^+^ (red), IgG^+^ (blue), and IgM^+^ (gray) cells in plasmablast and memory B cell populations at day 10 and day 30 post-immunization. (**D**–**F**) The t-SNE plots show expression levels of genes ITGA4 (left panel) and ITGB1 (right panel) (**D**), CCR10 (**E**), and J CHAIN (**F**) in B cells on days 10 and 30 after intranasal vaccination. Color intensity represents the level of expression. (**G**–**I**) Violin plots showing transcription expression levels of α4β1 (**G**), CCR10 (**H**), and J CHAIN (**I**) in specific B cell types on day 10. Statistical significance was determined using the wilcoxon test, ****P < 0.0001. (**J** and **K**) Pie charts depicting the relative proportions of IgA^+^ (red), IgG^+^ (blue), and IgM^+^ (gray) cells in α4β1^+^ (**J**) and CCR10^+^ (**K**) plasmablast populations on day 10. (**L**) B cells expressing the same clonotypes as the 45 nasal mucosal mAbs in day 10 and day 30 single B cell sequencing data. Clonotypes 719-3, -4, -17, -30, -33, -39, and -43 were present in the IgA isotype (left panel), while clonotypes 719-3, -4, -11, and -45 were present in the IgG isotype (right panel).

The tissue-specific homing of lymphocytes depends on the expression patterns of their homing receptors. It remains unclear how the intranasal booster induces localized mucosal sIgA production by modulating the homing properties of peripheral lymphocytes. To understand the phenotypic characteristics of B cells after the intranasal booster, we performed scRNA-seq on CD27^+^ B cells isolated from PBMCs of the same donor on days 10 and 30 following the first intranasal booster (Figure 4A and Supplemental Table 4). On day 10, four distinct B cell subtypes or states were identified, including memory B cells, plasmablasts, naive B cells, and exhausted B cells. By contrast, plasmablasts were nearly undetectable by day 30 (Figure 4B). Plasmablasts represent an antigen-induced transitional state of activated B cells. On day 10, 56.2% of plasmablasts expressed IgA, 11.7% expressed IgG, and 13.2% expressed IgM, indicating that intranasal booster predominantly promoted IgA^+^ B cell responses (Figure 4C). In contrast, 28.2% of memory B cells expressed IgA, 24.9% expressed IgG, and 46.3% expressed IgM by day 30 (Figure 4C). On day 10, plasmablasts express high levels of integrin α4 (ITGA4) and integrin β1 (ITGB1), which form the α4β1 integrin that can bind to vascular cell adhesion molecule-1 (VCAM-1) on mucosal epithelial cells (Figure 4, D and G). These plasmablasts also express high levels of the chemokine receptor 10 (CCR10), which is critical for homing to the nasal mucosa (Figure 4, E and H). J chain expression was the highest in plasmablasts (Figure 4, F and I). In total, 69.4% of ITGA4^+^ITGB1^+^ plasmablasts and 73.1% of CCR10^+^ plasmablasts express IgA, indicating IgA^+^ plasmablasts have upregulated expression of mucosal homing molecules (Figure 4, J and K, and Table 4). Searching for the same clonotypes as the mucosal-derived mAbs in scRNA-seq revealed 7 B cell clonotypes in the IgA isotype: 719-3, 719-4, 719-17, 719-30, 719-33, 719-39, and 719-43. Fewer B cell clonotypes (719-3, 719-4, 719-11, and 719-45) in IgG isotype could be found, and the numbers were also much less than those of the IgA isotypes (Figure 4L). These results demonstrated that the IgA class-switching, clonal expansion, and homing receptor upregulation of antigen-specific B cells occurred after the intranasal booster.

### Intranasal booster induces a transient elevation of cytokines and chemokines that facilitate B cell recruitment and antibody production in the nasal mucosa.

**Figure 5:**
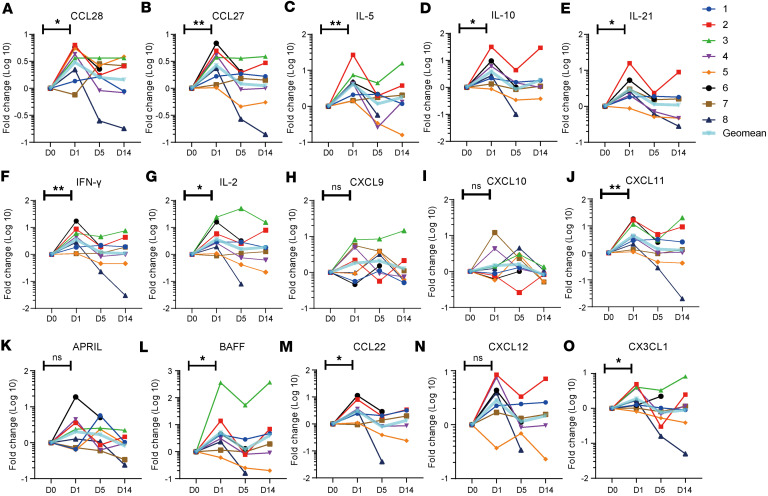
Cytokine and chemokine responses in the nasal passage after intranasal booster. (**A**–**O**) Nasal swab samples were collected from 8 donors before intranasal booster, and on days 1, 5, and 14 after intranasal vaccination. The expression levels of 15 cytokines and chemokines were measured using a Luminex multiplex assay. The data are expressed as fold changes relative to prevaccination cytokine and chemokine levels. Significance was determined by wilcoxon matched-pairs signed rank test, *P < 0.05; **P < 0.01.

To understand if intranasal booster induces changes in cytokines and chemokines in the nasal mucosa, which may provide insights into the induction of antigen-specific sIgA in the nasal mucosa, we collected nasal swabs from 8 volunteers on days 0, 1, 5, and 14 after the intranasal booster (Supplemental Table 6). A panel of 15 cytokines and chemokines associated with immune cell recruitment and homing was measured (Figure 5). There was a significant transient increase of C-C motif chemokine ligand 27 (CCL27), CCL28, IFN-γ, IL-2, IL-5, IL-10, IL-21, C-X-C motif chemokine ligand 11 (CXCL11), B cell activating factor (BAFF), C-X3-C motif chemokine ligand 1 (CX3CL1), and CCL22. CCL27 and CCL28 bind to CCR10 on B cells and promote the recruitment of IgA-secreting cells to the nasal mucosa (16). IFN-γ is associated with the induction of chemokines, such as CXCL11, which can promote Th1 cell recruitment (15, 25). IL-21 synergizes with TGF-β1 to promote class switch recombination (CSR) toward IgA (26, 27). IL-2, IL-5, and IL-10 promote the expansion of IgA class-switched B cells and their differentiation into IgA-secreting cells (27, 28). BAFF can promote B cell survival and antibody production (29). CCL22 promotes affinity maturation of B cells by facilitating positive selection in germinal centers (30).

## Discussion

Although the important role of respiratory mucosal sIgA induced by intranasal boosters against respiratory pathogens like SARS-CoV-2 has been generally recognized, the mechanisms by which an intranasal booster induces and regulates antigen-specific sIgA production remain poorly understood. This study provides insights into how intranasal booster elicits antigen-specific sIgA secretion in the nasal mucosa, a critical component in establishing the frontline immune defense against incoming respiratory pathogens, such as SARS-CoV-2. For the first time in the field of mucosal research, we obtained 42 functional human nasal mucosa-associated spike-specific sIgA mAbs by the combination of MS for sIgA peptide identification and scBCR-seq. Using deep sequencing of BCR repertoires, we were able to track the evolution of these spike-specific mAb clonotypes in the BCR reservoirs before and after each vaccination. Therefore, we provided evidence that an intranasal booster can restimulate spike-specific memory B cells primed by prior intramuscular vaccination to undergo clonal expansion, IgA class-switching, SHM, and homing to the nasal mucosa.

This study demonstrated that mucosal-derived sIgA mAbs possess superior potency than their IgG and IgA isotypes, up to 100-fold for some mAbs in neutralizing some SARS-CoV-2 variants. The hierarchy of neutralizing potency for these mAbs — i.e., sIgA/dIgA > IgG/mIgA — is consistent with our study using purified nasal sIgA, serum IgG, and IgA (6, 31). An earlier study showed that several RBD-specific IgA mAbs cloned from circulating B cells were converted to dIgA and demonstrated a 15-fold geometric mean increase in potency when neutralizing WT or ancestral SARS-CoV-2. However, they were not tested against Omicron variants (32). Another study converted 4 RBD-specific IgG to sIgA and found that 1 of them, mAb DXP-604, exhibited 3.5-, 26.3-, and 71.5-fold enhancements in neutralizing WT, BA.1, and BA.5, respectively (19). Apart from limited studies on the conversion of RBD-targeted IgG mAbs to sIgA, there have been no studies on nasal mucosal sIgA mAbs regarding their neutralizing potency, binding epitopes, and germline usage. In this study, the identification of 45 nasal mucosal spike-specific sIgA mAbs provides insights into the diversity and functional properties of nasal mucosal antibodies. These mAbs target a variety of epitopes, including NTD and different regions of RBD on the spike protein, and are derived from a variety of IgH and IgL germline genes.

The structural advantage of sIgA, with greater size, wider arm distance, and greater avidity, may explain why the RBD-targeting 719-14 and 719-40 sIgA mAbs achieved broad and potent neutralizing activity against even lately emerged Omicron subvariants XBB, JN.1, and KP.3. In contrast, their IgG counterparts were 1–2 orders of magnitude weaker against these Omicron subvariants. This finding underscores the critical or indispensable role of sIgA antibodies in the mucosa, which exhibit augmented neutralizing and binding potency compared with their IgG and IgA isotypes in the blood. This enhanced functionality is crucial for safeguarding the site of viral invasion. Concurrent with this finding, it was observed that certain mAbs, including NTD-targeted 719-19 and 719-39, when expressed as IgG isotypes, exhibited minimal neutralizing activity against BA.1 and KP.3, whereas their sIgA isotypes exhibited potent neutralizing activities against these variants. The presence of 4 or more paratopes on sIgA that drives epitope recognition may offer enhanced affinity, avidity, and other indirect effects that allow direct neutralization and steric hindrance in the form of sIgA rather than monomeric IgG or IgA. Thus, mucosal sIgA has the advantage of interfering with the conformational epitopes critical for viral binding and entry into cells, making it evolutionarily optimally suitable for the frontline defense in upper respiratory tract.

Longitudinal analysis of the BCR repertoires revealed the dynamics of circulating B cells from the naive state (before vaccination) to the postintramuscular and postintranasal vaccination states. After intramuscular vaccination, the B cells expressing the same antibody clonotype as those mucosa-associated mAbs were found in IgM, IgG, and IgA isotypes. After intranasal booster, the antibody clonotypes were mostly present as IgA isotype with less IgG isotype. Therefore, after intranasal booster, the expansion of spike-specific IgA^+^ B cells was mainly the result of intranasal booster restimulated IgG^+^ memory B cells and class switching to IgA^+^ B cells, accompanied with increased SHM. Interestingly, The decreased spike-specific IgA^+^ B cells in the BCR reservoir at 3 months after intranasal booster coincides with the observation that the level of nasal spike-specific sIgA declined about 65% but remained at a high level at 3 months after intranasal booster (11). Another study showed that nasal spike-specific sIgA wanes 9 months after a natural infection (33). These observations suggest that repeated intranasal boosters may be needed regularly to maintain high levels of mucosal sIgA for protection against respiratory infections. Although the decline in nasal sIgA level over time highlights the need for strategies to enhance durability, the persistence of antigen-specific IgA^+^ memory B cells in BCR repertoires suggested the capability for rapid recall responses by a new intranasal booster.

Of note, the IgH sequences obtained at each time point, containing approximately 5 million PBMCs, represent only a small fraction of the total B cell repertoire in the body — i.e., 0.03%–0.3% — assuming there are 1 × 10^7^ to 1 × 10^8^ unique B cell clonotypes in an adult. Therefore, B cell clonotypes in low numbers may not be collected. The 719-3 clonotypes were initially detected in IgM and IgG isotypes, but not in IgA, following the first intramuscular vaccination. Notably, 2 weeks after the first intranasal booster, a substantial number of 719-3 clonotypes in IgA appeared, indicating marked IgA class-switching. These IgA clonotypes had higher rates of SHM than the IgG isotype. Therefore, after intranasal booster, the expansion of spike-specific IgA^+^ B cells was mainly resulted from intranasal booster restimulated IgG^+^ memory B cells and class switching to IgA^+^ B cells, accompanied with increased SHM. Although the 719-3 antibody neither binds nor neutralizes the WT, the processes of class switching and affinity maturation may have subsequently conferred upon it the ability to bind the Omicron variant. In this study, among 7 mucosa-associated mAbs of which their clonotypes could be found in the BCR repertoires, 3 clonotypes, 719-16, 719-25, and 719-40, were found in the BCR repertoires postintranasal booster and were found only in IgA but not in IgG or IgM isotype. There are 2 possible reasons why these clonotypes were not found in the BCR repertoires after intramuscular priming: (a) the number of IgM^+^ and IgG^+^ B cells expressing these clonotypes were in too small number in the circulation and were not present in the 5 million PBMCs that we collected and sequenced, and (b) these clonotypes may have originated from a de novo B cell response induced by the intranasal booster, rather than from reactivation of preexisting memory B cells. Therefore, it is possible that intranasal vaccination can also elicit primary B cell response, alongside with the restimulation of memory B cells, in shaping the mucosal antibody repertoire.

ScRNA-seq revealed that these expanded B cells, especially IgA^+^ plasmablasts and memory B cells, exhibit upregulated expression of mucosal homing molecules, such as CCR10 and α4β1. Concomitantly, the intranasal booster induced a significant elevation of homing-related cytokines and chemokines, such as CCL28 and CCL27, in the nasal mucosal environment, which is likely crucial for attracting and retaining the IgA^+^ B cells to the nasal mucosal site. The kinetics of cytokine production reflect a coordinated immune response. Th1 cell-derived IFN-γ promotes cellular immunity, while BAFF, IL-5, IL-10, and IL-21 promote B cell survival, IgA class switching, and antibody production in the nasal mucosa.

We noted that a live-virus neutralization assays would directly reflect protective efficacy of COVID-19 vaccination. However, the neutralization assays with live SARS-CoV-2 must be carried out in a qualified Biosafety Level 3 (BSL-3) lab, which is currently unavailable in our institute. Nevertheless, the pseudovirus neutralizing antibody responses may be used as surrogates for predicting protective efficacy. Many studies have shown that the neutralizing antibody titers measured by the lentivirus-based pseudoviruses, which was also used in our study, are well correlated with the protective efficacy of COVID-19 vaccines in both animal models and humans (34–37).

Our study has several limitations. The discovery of 45 mucosa-associated mAbs and the associated analysis of the BCR repertoire and transcriptome were all derived from a single donor, which may restrict the generalizability of the findings. Additionally, while the observed cytokine and chemokine milieu is consistent with a mucosal immune response, these cellular data were derived from peripheral blood. The anatomical sites where SHM and class switching occurred remain to be elucidated. Future studies should investigate whether IgA^+^ B cells are generated or reactivated in the NALT; cervical lymph nodes, which serve as the major draining nodes of the upper respiratory tract; or other mucosa-associated structures.

Our findings have marked implications for mucosal vaccine design. The failure of intramuscular vaccines to induce nasal mucosal sIgA left a gap in preventing infection and transmission. Our data support the need for prioritizing the development of mucosal vaccines, particularly intranasal platforms to establish local mucosal immunity. A prime-boost strategy — e.g., systemic prime followed by intranasal booster as seen in our studies — may optimally engage both compartments, where intramuscular vaccines seed memory B cells that later differentiate into IgA^+^ effectors upon intranasal booster. This approach could be critical for combating rapidly evolving pathogens, where the ability of mucosal sIgA antibodies that can cover broader epitopes may mitigate viral escape. Additionally, the diversity of sIgA mAb epitopes supports the inclusion of full-length spike or multivalent antigens in mucosal vaccines to maximize neutralizing breadth. In an earlier report (31), we found that nasal sIgA from BA.5 convalescents who previously received the injected whole-virus vaccine had greater neutralizing potency (IC_50_ 10 nM against WT, 5 nM against BA.5) than vaccine-naïve convalescents (IC_50_ 893 nM against WT, 21 nM against BA.5). Therefore, a prior intramuscular vaccination may provide antigen-specific memory B cells that the intranasal booster can lead to rapid activation and homing to the nasal mucosal to produce sIgA.

In summary, this work provided insights for how intranasal booster generates potent mucosal sIgA responses. These findings enforce the idea that nasal vaccines should confer protection against respiratory pathogens at their point of entry. As the SARS-CoV-2 pandemic highlights the limitations of systemic immunity alone, embracing nasal vaccination could enhance our ability to curb transmission and prevent future X pandemics, which are more likely transmitted via airway. Future efforts should explore whether intranasal vaccination alone or a combinatorial approach — i.e., pairing intramuscular priming with the intranasal booster — is more rapid and effective in preventing infections of respiratory viruses, especially rapidly evolving viruses such as SARS-CoV-2, influenza viruses, and future X respiratory pathogens.

## Methods

### Sex as a biological variable.

For antibody purification, nasal mucosal lining fluids (NMLF) and peripheral blood were collected from 3 male and 3 female volunteers, whereas for cytokine or chemokine analysis, NMLF was collected from 6 male and 2 female volunteers.

### Intranasal vaccination and sample collection.

Human intranasal vaccination was performed according to the manufacturer’s protocol (TZ-BN30, Tianzhou packing). In brief, 0.2 mL of NB2155 vaccine (containing 2 × 10^10^ viral particles of Ad5-S-Omicron) was drawn into a syringe fitted with a nasal spray applicator. The applicator tip was firmly positioned against the nostril, and the vaccine was administered via a swift compression of the device. This process was then replicated in the contralateral nasal cavity. Peripheral blood was collected using vacuum tubes, and PBMCs were isolated for sequencing. Serum samples were collected and heat-inactivated at 56°C for 30 minutes. NMLF was harvested either through nasal swabs with subsequent elution of samples into 1.0 mL of saline for cytokine and chemokine analysis or via nasal lavage using a nasal wash irrigator with 200–300 mL of saline for antibody purification purposes. Protein concentration within the NMLF sample was quantified using a bicinchoninic acid assay (BCA) protein assay kit (Thermo Fisher, USA).

### Expression of nasal mucosal mAbs.

The variable region genes were synthesized and cloned into IgG1 and IgA1 expression plasmids (refer to as IgG and IgA thereafter). The light and heavy chain plasmids at a 1:1 ratio were transfected into Expi293F cells (A14527CN, Thermo Fisher, USA) by EZ Trans Cell Transfection Reagent (AC04L092, Life-iLab). For dIgA1 and sIgA1, the J chain plasmids only, or J chain and SC plasmids, were added. Cell culture media were harvested at 6 days after transfection for purification of mAbs.

### Antibody purification.

Serum samples were diluted 10-fold in DPBS. NMLF was concentrated using a 100 kDa molecular weight cutoff (MWCO) centrifugal filter (Merck Millipore). Supernatants containing recombinant mAbs were clarified by centrifugation at 10,000 × *g* and subsequent filtration through 0.45 μm filters. Serum IgG and monoclonal IgG were purified by affinity chromatography on Protein G Resin (GenScript). Nasal, serum, and monoclonal IgA were purified using peptide M-coupled agarose beads (InvivoGen). Purified antibodies were concentrated using a 100 kDa MWCO ultrafiltration device (Merck Millipore), and the buffer was exchanged to DPBS. The concentration of purified antibodies was determined spectrophotometrically by measuring absorbance at 280 nm with a Nanodrop 8000 instrument (Thermo Fisher). Purified antibodies were then employed for downstream binding and neutralization assays.

### Fractionation of recombinant monoclonal dIgA and sIgA by size exclusion chromatography.

The Superdex 200 Increase 10/300 GL gel filtration column (Cytiva) integrated with the Bio-Rad FPLC AKTA Chromatography System was calibrated at ambient temperature. Calibration was performed with the high molecular weight (HMW) Gel Filtration Calibration Kit (Cytiva) and IgG. Following column equilibration with DPBS, purified monoclonal dIgA or sIgA were loaded via a 0.5 mL loop at a 0.5 mL/min flow rate. Nasal IgA polymers, dimers, and monomers were resolved based on quaternary structure. Series fractions of 0.4 mL were collected in DPBS at a 0.5 mL/min flow rate. Subsequently, fractions were analyzed via SDS-PAGE under nonreducing conditions, followed by Coomassie blue staining and Western blot analysis. The fractions consisting mainly of dimers were pooled and concentrated for subsequent analysis.

### Luminex multiplex cytokine assay.

Nasal swab samples were collected before vaccination and on days 1, 5, and 14 after vaccination. Cytokines and chemokines were measured for each donor in nasal swab samples using multiplex cytokine assays (PPX-17-MXKA5MZ, LXSAHM-04) on the Luminex 200 system performed at the Laizee Biotech, Shanghai, China. Individual data were normalized to equal amounts of total IgA. The data are expressed as fold changes relative to prevaccination cytokine and chemokine levels. Significance was determined by Wilcoxon matched-pairs signed rank test, with *P* < 0.05 considered significant.

### Neutralization assays with lentivirus-based pseudoviruses.

Neutralization assays were performed using lentivirus-based pseudoviruses. Antibodies were serially diluted in DMEM, starting at a concentration of 750 nM and using 6 consecutive 1:3 or 1:4 dilutions. A 50 μL aliquot of each dilution was incubated with 50 μL of pseudovirus supernatant containing 800 50% tissue culture infective dose (TCID_50_) in a white 96-well cell culture plate (WHB, China) for 1 hour at 37°C. Subsequently, 20,000 ACE2-293T cells in 100 μL were added to each well. Following a 72-hour incubation period at 37°C and 5% CO_2_, the reduction in relative luminescence units (RLU) was quantified in virus-infected vs. untreated control cells. Luminescence was measured using the Bio-LiteTM Luciferase Assay System (Vazyme, China) according to the manufacturer’s protocol. The IC_50_ was determined using the Reed-Muench method.

### SDS-PAGE and Western blot analysis.

SDS-PAGE and Coomassie blue staining were performed using 4 μg of antibodies. For Western blot analysis, 200 ng of antibodies were separated by SDS-PAGE and transferred to PVDF membranes. Commercially available sIgA derived from colostrum, serum IgA, and serum IgG were included as reference standards. Following the transfer, membranes were incubated with horseradish peroxidase–conjugated (HRP-conjugated) goat anti-human IgA α chain antibody (ab97215, Abcam, UK) or goat anti-human IgG (H+L) antibody (A0201, Beyotime, China). Chemiluminescence was detected using a HRP substrate and a Bio-Rad imaging system.

### BLI.

Competitive binding assays were performed using a Gator label-free bioanalysis system (GatorBio). All experiments were carried out at room temperature with shaking (Shaker A: 400 rpm; Shaker B: 1000 rpm). A black 96-well microplate was sequentially added with 5 μg/mL recombinant SARS-CoV-2 WT RBD protein (Sino Biological), Q buffer, 10 μg/mL test antibody, and 10 μg/mL known standard antibodies or ACE2. The Gator instrument was programmed with the following steps: baseline (120 sec), loading (120 sec), association 1 (300 sec), and association 2 (300 sec). The SARS-CoV-2 RBD was loaded onto HIS1K biosensors, and the RBD-coated sensors were saturated with the recombinant mAbs before incubation with the known antibodies or ACE2. Binding kinetics were analyzed using Gator System Analysis software.

### ELISA.

ELISA was conducted to assess the binding of antibodies to the SARS-CoV-2 spike protein. Ninety-six well plates were coated with 100 ng of S protein (Sino Biological) and incubated overnight at 4°C. Plates were then blocked with 1× DPBS supplemented with 5% skim milk for 2 hours at 37°C. Serial 3-fold dilutions of purified antibodies were added to the wells, starting at concentrations of 125 μg/mL for serum antibodies, 37.5 μg/mL for nasal sIgA, and 4-64 nM for mAbs. Following a 2-hour incubation at 37°C, wells were incubated with HRP-conjugated goat anti-human IgA α chain antibody (ab97215, Abcam, UK) or goat anti-human IgG Fc antibody (ab97225, Abcam, UK). After 1 additional hour at 37°C, the reaction was developed using 3,3’,5,5′-tetramethylbenzidine (TMB) substrate, and absorbance was measured at 450 nm. EC_50_ values were determined using the 4-parameter nonlinear regression analysis (GraphPad Prism).

### LC-MS/MS for peptide identification of spike-binding sIgA.

Nasal sIgA was digested with an immobilized pepsin resin (Thermo Fisher) to prepare F(ab)2. Biotin-labeled BA.1 S protein (Sino Biological) was coincubated with F(ab’)2 on a shaker at 4°C for 12 hr and then incubated with streptavidin magnetic beads (Thermo Fisher) on a shaker at room temperature for 2 hr. The magnetic beads were washed 3x with DPBS to remove unbound proteins. The spike-specific sIgA was eluted with Gly-HCl at pH 1.7 and then neutralized with Tris-HCl at pH 7.5. The eluted fractions were desalted using a Zebra desalting column (Thermo Fisher). ELISA was used to detect spike-specific sIgA enrichment in the eluted fractions.

The enriched samples were denatured in 2,2,2-trifluoroethanol (TFE), 50 mM ammonium bicarbonate, and 10 mM dithiothreitol (DTT) for 1 hr and then alkylated with 32 mM iodoacetamide (Sigma-Aldrich) for 30 min in the dark at room temperature. The samples were then digested with trypsin at 37°C for 16 hr. Formic acid was added at 1% (v/v) to quench digestion. Following trypsin digestion, peptides were purified using the HyperSep Spin Tip C-18 kit (Thermo Fisher). The samples were analyzed using LC-MS/MS using an Easy-nLC 1200 connected online to an Orbitrap Eclipse mass spectrometer (Thermo Fisher). A 250 mm Acclaim PepMap100 C18 column (Thermo Fisher) with internal diameter of 75 μm was used to separate the peptides with mobile phase A (0.1% FA in water) and mobile phase B (0.1% FA in 80% ACN) at a 60 min gradient: 4%–20% B in 45 min, 20%–36% B in 8 min, 36%–100% B in 2 min, and then maintained B at 100% B for 5 min. The flow rate was set at 300 nL/min. The Orbitrap Eclipse mass spectrometer was operated in the data-dependent acquisition mode. MS1 data were collected using an Orbitrap (60,000 resolution; AGC target 100%; maximum injection time 50 ms). Charge states between 2 and 7 were required for sequencing, and a 50 s dynamic exclusion window was used. The MS2 stage consisted of fragmentation by HCD (normalized collision energy 30%) and analysis using Orbitrap (15,000 resolution, AGC target 100%, maximum injection time 22 ms, isolation window 1.6 m/z). FAIMS voltage was set as −45 V and −65 V, and the cycle time was set at 1 s per CV. Raw LC-MS/MS data were obtained in the form of mass/charge ratio, retention time, and intensity. The raw data were decoded into peptides using Proteome discoverer software and Peaks Studio software. The peptides were then aligned with single BCR sequences obtained from PBMC of the same donor.

### Sequencing of BCR transcripts.

PBMC were isolated from a donor at different time points as follows: before vaccination (before Vx), 3 weeks after vaccination with the inactivated vaccine (3 weeks after i.m. Vx), 2 weeks after vaccination with the second inactivated vaccine (2 weeks after 2 i.m. Vx), 2 weeks after the first intranasal booster (2 weeks after 1 i.n. Vx), 2 weeks after the second intranasal booster (2 weeks after 2 i.n. Vx), and 3 months after second intranasal booster (3 mon after 2 i.n. Vx). Total RNA was extracted from 5 million PBMCs using TRIZOL reagent according to the manufacturer’s protocol (Life Technologies). VH transcripts were amplified using the dam-PCR technology (iRepertoire, USA) as described previously (38). Briefly, multiplex primers covering the human IGHV genes (forward primers) and constant region primers (reverse primers) were designed. The forward primer Fi (forward-in) and reverse primer Ri (reverse-in) also included Illumina paired-end sequencing communal primers B and A, respectively (Illumina, USA). Unique barcodes were introduced in the first round using constant region primers to distinguish the samples. After gel purification using a QIAquick gel extraction kit (Qiagen, Germany), the resulting product was pooled and sequenced on Illumina NovaSeq 6000 with paired-end 250 bp read mode (Novogene, China).

### Bulk RNA-seq analysis.

Sequences that share the same IGHV and IGHJ gene segments, identical HCDR3 length, and ≥ 75% HCDR3 identity were defined as a clonotype. Scatter plots were generated using ggplot2 in R to visualize antibody clonotype abundance in circulating B cells before and after vaccination. Sequence alignment and phylogenetic analysis of clonotypes were conducted in MEGA11 using the maximum likelihood method for phylogenetic tree construction.

### Single-cell 5′ mRNA and V(D)J sequencing and analysis.

B cells were isolated from the donor’s PBMCs by flow cytometric sorting of CD3^–^CD19^+^cells, CD3^–^CD19^+^CD27^+^cells, CD3^–^CD19^+^IgG^+^cells, or CD138^+^cells. The following anti-human monoclonal antibodies were used: Pacific Blue-CD3 (clone: SP34-2, BD Biosciences, USA), FITC-CD19 (clone: HIB19, BD Biosciences, USA), BB700-CD27 (clone: M-T271, BD Biosciences, USA), PE-IgG (clone: MOPC-21, BD Biosciences, USA), and BV421-CD138 (clone: MI15, BD Biosciences, USA). Sorted cells were sent for 5′-mRNA and V(D)J library preparation. Single-cell 5′-mRNA libraries were prepared using SeekOne DD Single Cell 5′ library preparation kit (SeekGene). Single-cell V(D)J libraries were prepared using SeekOne DD Single Cell 5′ library preparation kit and SeekOne DD Single Cell V(D)J Enrichment Kit (SeekGene, K01201). Libraries were then sequenced on Illumina NovaSeq 6000 with PE150 read length.

Raw sequencing data were analyzed using SeekSoul Tools and aligned against the IMGT BCR database to generate downstream datasets. 5′ mRNA transcriptome raw data were aligned against GRCh38. Cells were quality controlled (nFeature RNA > 200, nCount RNA < 10,000, and percent.mt < 10) using the R package Seurat, from which B cells were selected using CD19/CD20(MS4A1)/CD27 as markers. A total of 2,000 highly variable genes identified by the Find Variable Features function in the Seurat package were used for principal component analysis–based (PCA-based) dimensionality reduction using Run PCA. Single-cell clustering of principal components (PC) 1–10 was visualized using t-SNE. Visualization of single gene expression levels was obtained using feature plot and vln plot. Cell subtypes were identified using the R-package Single R.

### Statistics.

The IC_50_ and EC_50_ values were determined by fitting the data to a 4-parameter nonlinear regression using GraphPad Prism. Spearman’s rank correlations were used to establish multiparameter associations. Correlation plots were generated using the GraphPad Prism software. Comparison between the expression levels of cytokine from different times was performed using the Wilcoxon matched-pair signed-rank test using GraphPad Prism. *P* < 0.05 was considered statistically significant. Images were assembled using Adobe Illustrator. The graphical abstract was drawn using BioRender.

### Study approval.

In this study, NMLFs and peripheral blood samples were collected from donors enrolled in a clinical trial evaluating an intranasal vaccine from the First Affiliated Hospital of Guangzhou Medical University. All participants were provided with written informed consent before enrollment. This study was approved by the Guangzhou Eighth People’s Hospital, Guangzhou Medical University (reference no. 202303240), and the First Affiliated Hospital of Guangzhou Medical University (reference no. 2022173). The study was registered with the Chinese Clinical Trial Registry (ChiCTR2300070346).

### Data availability statement.

The data in this article are provided in the article, supplemental material, and Supporting Data Values file. The bulk sequencing data and the single-cell B cell transcriptome and VDJ sequencing data generated in this study have been deposited in the Genome Sequence Archive for Human (GSA-Human) under the accession no. HRA013311.

## Author contributions

SC designed the study and methodology and analyzed data. Z Lin performed bioinformatic analysis. ZZ performed Western blot, size exclusion chromatography, antibody expression, and antibody purification. LY performed the flow cytometry assay. LN, WL, YW, H Li, Y Liu, and BL conducted competitive binding assays, pseudovirus neutralization assay, and ELISA. QW, CY, BF, YF, PH, H Liang, and Z Li provided key reagents. Y Li, DB, and LQ reviewed and helped to improve the manuscript. SC and LC drafted the manuscript. LC, PL, NZ, and XN supervised the overall research project and reviewed and revised the manuscript. All authors have read and approved the final manuscript.

## Funding support

- National Natural Science Foundation of China (92469301, 32500826)
- National Key Research and Development Program of China (2024YFA0920001)
- Basic Research Project of Guangzhou Institutes of Biomedicine and Health (GIBHBRP24-02)
- Major Project of Guangzhou National Laboratory (GZNL2023A01009, GZNL2025A01004)
- Youth Innovation Promotion Association of CAS (2022361)
- State Key Laboratory of Respiratory Disease (SKLRD-Z-202208, SKLRD-Z-202106)

## Supplementary Material

Supplemental data

Unedited blot and gel images

Supporting data values

## Figures and Tables

**Table 1:**
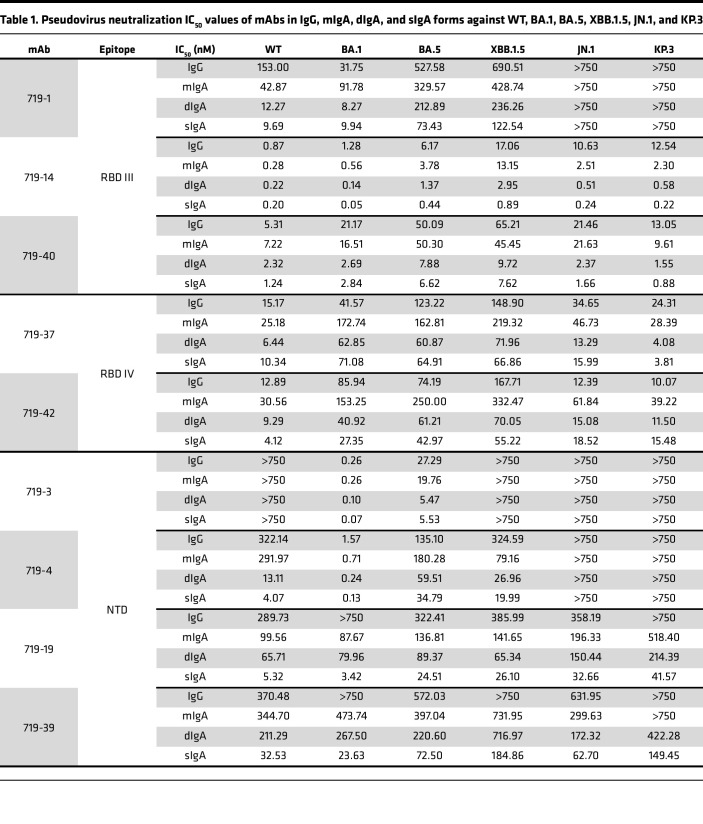
Pseudovirus neutralization IC_50_ values of mAbs in IgG, mIgA, dIgA, and sIgA forms against WT, BA.1, BA.5, XBB.1.5, JN.1, and KP.3

**Table 2:**
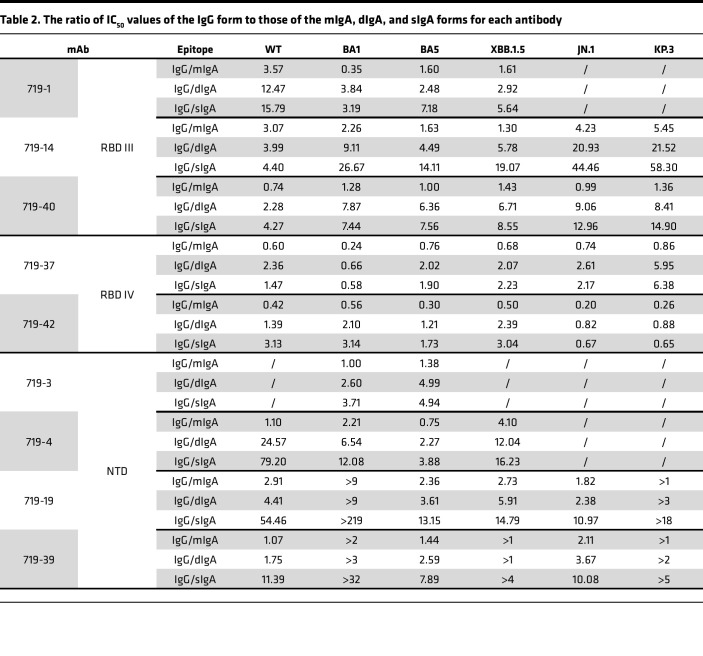
The ratio of IC_50_ values of the IgG form to those of the mIgA, dIgA, and sIgA forms for each antibody

**Table 3:**
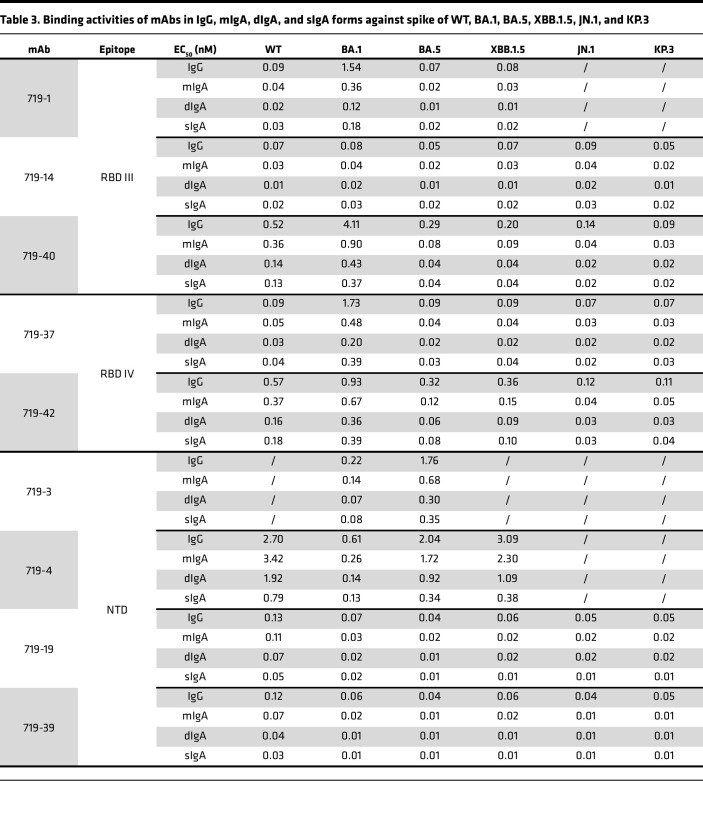
Binding activities of mAbs in IgG, mIgA, dIgA, and sIgA forms against spike of WT, BA.1, BA.5, XBB.1.5, JN.1, and KP.3

**Table 4:**
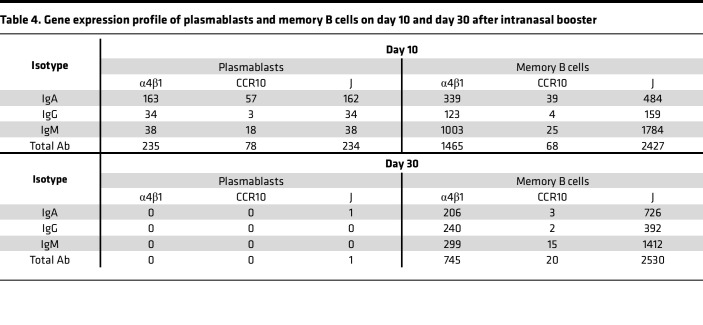
Gene expression profile of plasmablasts and memory B cells on day 10 and day 30 after intranasal booster

## Version 1 12/23/2025

In-Press Preview

## Version 2 02/09/2026

Electronic publication
