# Supplementary material for: Intranasal booster drives class switching and homing of memory B cells for mucosal IgA response
Source: JCI Insight. 2025 Dec 23;11(3):e198045. doi: 10.1172/jci.insight.198045 (PMC12892890; doi:10.1172/jci.insight.198045)
Supplement: Supplemental data [file jciinsight-11-198045-s159.pdf]

Supplemental material

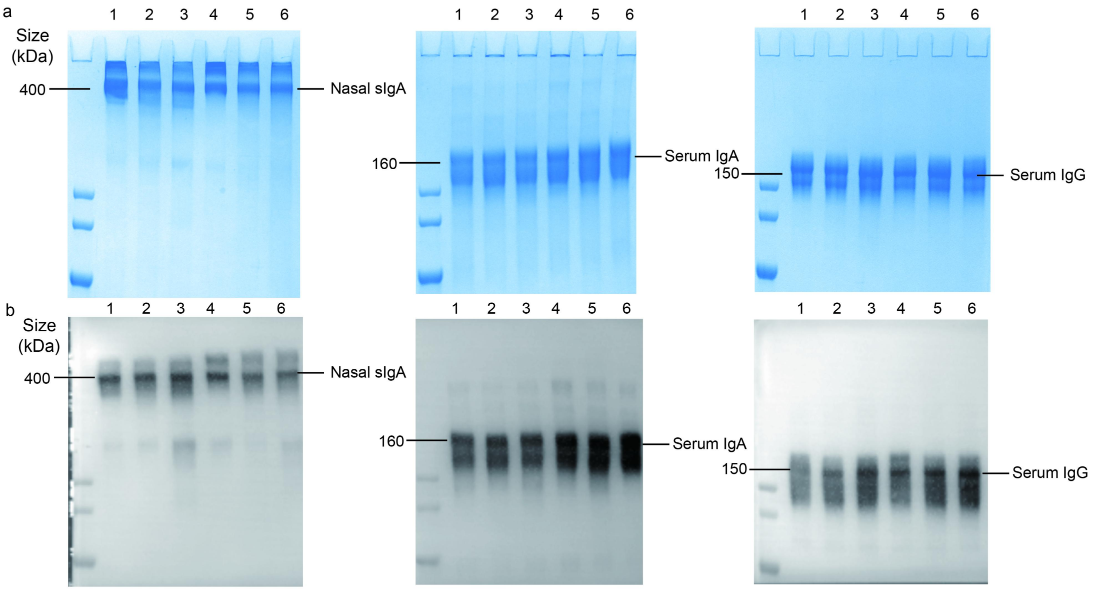

Supplementary Figure S1. SDS–PAGE and Western blot analysis of purified nasal sIgA, serum IgA, and serum IgG from six donors after intranasal booster.

a-b. Paired nasal sIgA, serum IgA, and serum IgG were purified from the same donors using affinity chromatography. Purified antibody samples were analyzed by non-reducing SDS-PAGE and Western blot. Anti-human IgA α-heavy chain (HCα) antibody and anti-human IgG H+L antibody were used for detecting IgA and IgG, respectively.

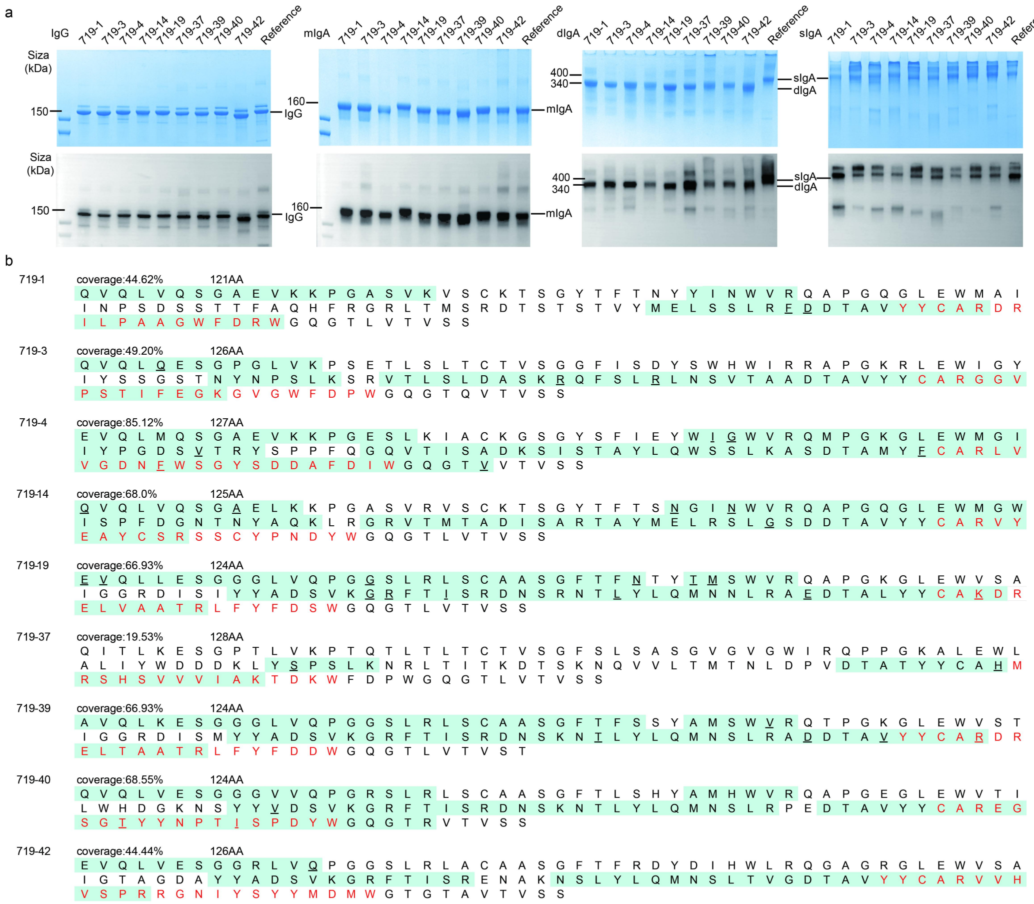

Supplementary Figure S2. The identification and expression of nasal mucosal mAbs.

a. Expression and purification of mAbs 719-1, 719-3, 719-4, 719-14, 719-19, 719-37, 719-39, 719-40, and 719-42 in IgG, mIgA, dIgA, and sIgA forms. The purity and molecular weight of each antibody were analyzed by non-reducing SDS–PAGE and Western blot.

b. Liquid chromatography-tandem mass spectrometry-detected peptides matching the heavy chain VJ gene and CDR3 sequences are highlighted in green, and CDR3 regions are marked in red.

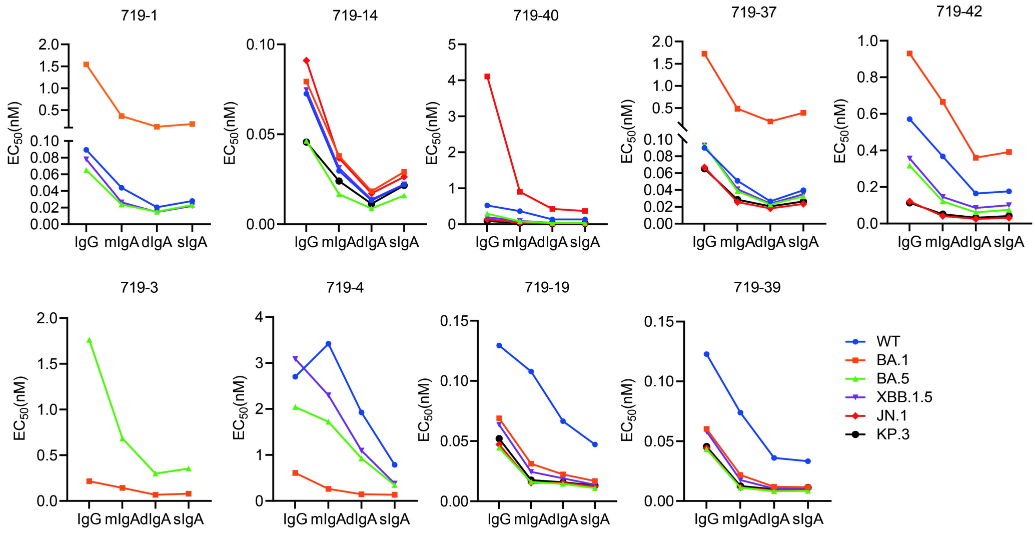

Supplementary Figure S3. The spike binding activities of nasal mucosal mAbs in IgG, mIgA, dIgA, and sIgA forms.

The binding activities of mAbs for the spike proteins of WT, BA.1, BA.5, XBB.1.5, JN.1, and KP.3 were measured by ELISA. The data are presented as half-maximal effective concentrations (EC_{50}).

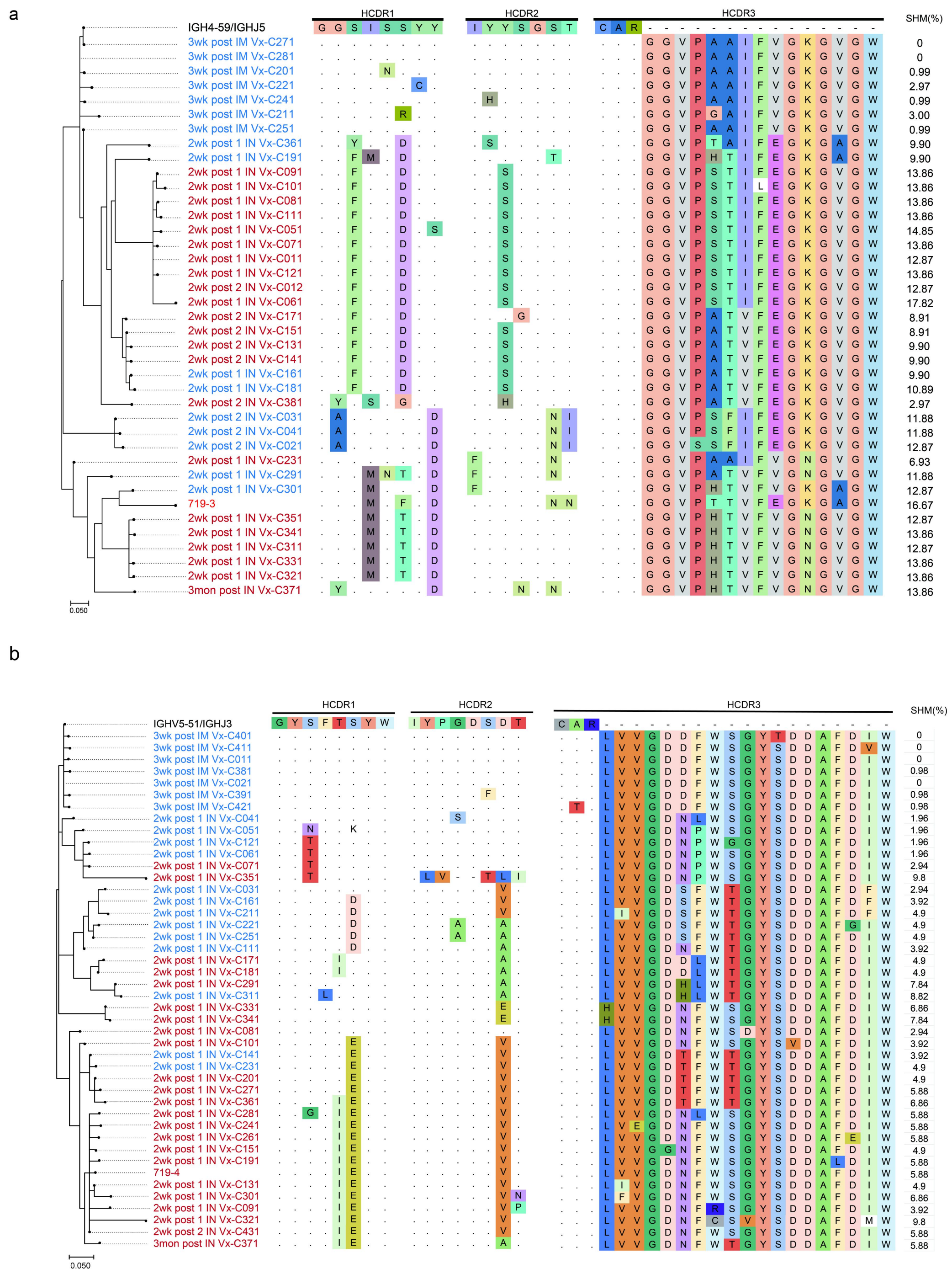

Supplementary Figure S4. Genealogical tree of 719-3 and 719-4 -like antibodies.

a. Genealogical tree of 719-3-like sequences descending from germline gene IGHV4-59/J5 for representative antibody clusters in the BCR repertoires at different time points after vaccination. Maximum-likelihood (ML) tree (left panel), multiple alignments of amino acid sequences (middle panel), and somatic hypermutation rates of each sequence (right panel) are shown. The antibody isotypes are color-coded: IgA is red, and IgG is blue.

b. Genealogical tree of 719-4-like sequences descending from germline gene IGHV5-51/J3 for representative antibody clusters in the BCR repertoires at different time points after vaccination. Maximum-likelihood (ML) tree (left panel), multiple alignments of amino acid sequences (middle panel), and somatic hypermutation rates of each sequence (right panel) are shown. The antibody isotypes are color-coded as IgA in red and IgG in blue.

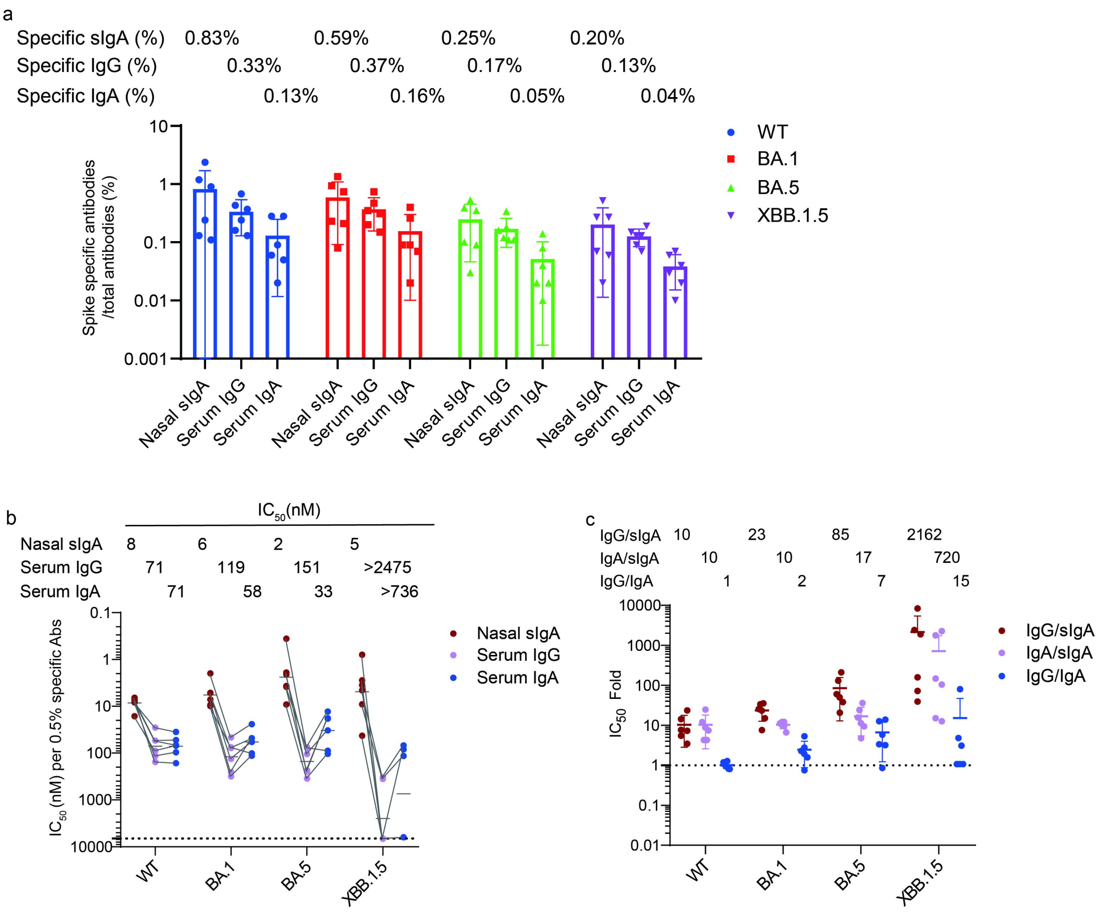

Supplementary Figure S5. Neutralization activity of nasal sIgA, serum IgA, and serum IgG after normalization to 0.5% spike-specific antibodies.

a. Estimation of spike-specific antibody proportions. The percentage of WT, BA.1, BA.5, and XBB.1.5 spike-specific antibodies within total nasal sIgA, serum IgG, and serum IgA was measured by ELISA, using mAb 719-1 (in the corresponding isotype) as a reference standard. Data are shown as mean ± SD (n = 6).

b. Neutralizing activity of nasal sIgA, serum IgA, and serum IgG after normalizing all samples to contain 0.5% spike-specific antibodies was assessed using a lentivirus-based pseudovirus system. The results are presented as 50% inhibitory concentration (IC_{50}) in nM. Samples showing no detectable neutralization activity at the highest concentration (1000 μg/mL) were assigned an IC_{50} value of 6666.7 nM (dashed line), representing no neutralization. Geomean values (n=6) are reported above the graph.

c. The ratios of IC_{50} between paired IgG/sIgA, IgA/sIgA, and IgG/IgA for each donor are presented. Mean values (n=6) are reported above the graph.

Supplementary Table S1. Demographics and vaccine regimens of the study cohort

Donor ID	Age (year)	Gender	Vaccine dose #1 Date (Intramuscular vaccination with inactivated vaccine)	Vaccine dose #2 Date (Intramuscular vaccination with inactivated vaccine)	Manufacturer	Vaccine dose #3 Date (Intranasal vaccination with NB2155)	Vaccine dose #4 Date (Intranasal vaccination with NB2155)	Manufacturer	Sample collection time (days post intranasal vaccination)
1	60	Male	8-Jul-2021	13-Aug-2021	SinovacBiotech	16-Mar-2022	27-Apr-2022	nBiomed	27
2	43	Female	31-Jul-2021	29-Aug-2021	Sinopharm	22-Nov-2022	22-Dec-2022	nBiomed	39
3	26	Male	7-Apr-2021	25-May-2021	SinovacBiotech	25-Nov-2022	28-Dec-2022	nBiomed	31
4	27	Female	13-Mar-2021	13-Apr-2021	SinovacBiotech	8-Dec-2022	15-Jan-2023	nBiomed	20
5	26	Male	12-Mar-2021	7-Apr-2021	SinovacBiotech	8-Dec-2022	15-Jan-2023	nBiomed	20
6	25	Female	19-May-2021	10-Jun-2021	Sinopharm	8-Dec-2022	15-Jan-2023	nBiomed	20

Supplementary Table S2. Pseudovirus neutralization and binding activities of serum IgG, serum IgA, and nasal sIgA from six donors against Omicron subvariants BA.1, BA.5, XBB.1.5 and pre-Omicron WT

IC_{50} (nM)

Serum IgG	Pseudovirus	1	2	3	4	5	6	Geomean
	WT	113.74	154.45	171.28	220.19	58.81	102.02	125.86
	BA.1	212.15	213.14	855.67	195.02	65.58	80.14	184.66
	BA.5	512.64	421.50	1056.75	1138.06	228.16	212.55	482.40
	XBB.1.5	6666.67	6666.67	6666.67	1871.49	6666.67	1188.10	4046.81
**Serum IgA**	**Pseudovirus**	**1**	**2**	**3**	**4**	**5**	**6**	**Geomean**
	WT	286.01	785.06	1441.22	652.70	61.81	404.98	417.39
	BA.1	214.99	403.55	978.52	285.53	30.19	531.89	270.21
	BA.5	630.89	716.09	968.48	367.70	45.27	1161.88	451.42
	XBB.1.5	6666.67	6666.67	6666.67	1368.22	624.76	1623.81	2727.02
**Nasal sIgA**	**Pseudovirus**	**1**	**2**	**3**	**4**	**5**	**6**	**Geomean**
	WT	2.85	38.58	15.17	29.39	4.72	3.52	9.66
	BA.1	5.18	33.04	17.51	11.70	1.38	3.82	7.55
	BA.5	13.74	32.96	20.15	11.04	0.47	3.57	7.43
	XBB.1.5	80.88	74.34	32.08	33.76	1.55	9.04	21.21

EC_{50} (nM)

Serum IgG	Spike	1	2	3	4	5	6	Geomean
	WT	83.40	51.88	143.47	166.47	34.99	16.40	62.45
	BA.1	415.27	87.87	317.93	172.33	96.93	71.07	154.82
	BA.5	154.00	182.33	183.07	53.68	24.33	17.51	69.99
	XBB.1.5	208.27	181.07	746.00	154.73	24.29	15.95	109.10
**Serum IgA**	**Spike**	**1**	**2**	**3**	**4**	**5**	**6**	**Geomean**
	WT	176.00	311.13	1785.00	337.13	40.53	159.63	244.42
	BA.1	409.13	430.81	1135.00	429.56	148.00	700.00	455.25
	BA.5	291.19	216.88	506.94	189.44	34.36	119.13	170.80
	XBB.1.5	1249.38	1318.13	1965.00	596.25	51.36	103.50	466.12
**Nasal sIgA**	**Spike**	**1**	**2**	**3**	**4**	**5**	**6**	**Geomean**
	WT	2.73	9.31	11.39	12.79	4.71	3.02	6.12
	BA.1	5.86	12.29	9.55	8.52	1.34	3.50	5.49
	BA.5	3.47	19.30	16.05	9.12	3.37	2.64	6.66
	XBB.1.5	6.37	24.50	40.36	26.47	4.35	3.66	11.77

Supplementary Table S3. The IC_{50} ratios and EC_{50} ratios of serum IgG to nasal sIgA, serum IgA to nasal sIgA, and serum IgG to serum IgA for each donor

IC_{50} Fold		1	2	3	4	5	6	Mean
serum IgG/nasal sIgA	WT	39.95	4.00	11.29	7.49	12.47	28.95	17.36
	BA.1	40.92	6.45	48.87	16.67	47.42	20.98	30.22
	BA.5	37.30	12.79	52.45	103.08	486.66	59.54	125.30
	XBB.1.5	82.42	89.68	207.79	55.43	4310.07	131.39	812.80

IC_{50} Fold		1	2	3	4	5	6	Mean
serum IgA/nasal sIgA	WT	100.46	20.35	94.98	22.21	13.10	114.90	61.00
	BA.1	41.47	12.21	55.89	24.40	21.83	139.22	49.17
	BA.5	45.91	21.73	48.07	33.30	96.56	325.45	95.17
	XBB.1.5	82.42	89.68	207.79	40.52	403.92	179.57	167.32

IC_{50} Fold		1	2	3	4	5	6	Mean
serum IgG/serum IgA	WT	0.40	0.20	0.12	0.34	0.95	0.25	0.38
	BA.1	0.99	0.53	0.87	0.68	2.17	0.15	0.90
	BA.5	0.81	0.59	1.09	3.10	5.04	0.18	1.80
	XBB.1.5	1.00	1.00	1.00	1.37	10.67	0.73	2.63

EC_{50} Fold		1	2	3	4	5	6	Mean
serum IgG/nasal sIgA	WT	30.55	5.57	12.60	13.02	7.43	5.43	12.43
	BA.1	70.87	7.15	33.29	20.23	72.34	20.31	37.36
	BA.5	44.38	9.45	11.41	5.89	7.22	6.63	14.16
	XBB.1.5	32.70	7.39	18.48	5.85	5.58	4.36	12.39

EC_{50} Fold		1	2	3	4	5	6	Mean
serum IgA/nasal sIgA	WT	64.47	33.42	156.72	26.36	8.61	52.86	57.07
	BA.1	69.82	35.05	118.85	50.42	110.45	200.00	97.43
	BA.5	83.92	11.24	31.59	20.77	10.20	45.13	33.81
	XBB.1.5	196.14	53.80	48.69	22.53	11.81	28.28	60.21

EC_{50} Fold		1	2	3	4	5	6	Mean
serum IgG/serum IgA	WT	0.47	0.17	0.08	0.49	0.86	0.10	0.36
	BA.1	1.02	0.20	0.28	0.40	0.65	0.10	0.44
	BA.5	0.53	0.84	0.36	0.28	0.71	0.15	0.48
	XBB.1.5	0.17	0.14	0.38	0.26	0.47	0.15	0.26

Supplementary Table S4. Collection time of PBMC samples from Donor 1 for 5' scRNA-seq & scV(D)J-seq(BCR)

Donor ID	Vaccination date (Intranasal booster with NB2155)	Manufacturer	PBMC collection date	Sample name	B cell types	Name	5' scRNA-seq	scV(D)J-seq(BCR)
Donor 1	16-Mar-22	nBiomed	26-Mar-22	10d post 1 IN Vx	CD138$^+$B	46383#1	7569	1533
		nBiomed			CD27$^+$B	46383#2	8713	4145
		nBiomed	19-Apr-22	30d post 1 IN Vx	CD27$^+$B	46383#3	8210	6334
	27-Apr-22	nBiomed	27-May-22	30d post 2 IN Vx	IgG$^+$B	46383#4	4243	3330
		nBiomed			CD27$^+$B	46383#5	3965	2677
	28-Jul-22	nBiomed	4-Aug-22	7d post 3 IN Vx	CD138$^+$B	46383#6	1157	864
		nBiomed	5-Dec-22	4m post 3 IN Vx	CD27$^+$B	46383#7	4010	3277
	8-May-24	nBiomed	15-May-24	7d post 4 IN Vx	CD19$^+$B	46383#8	12875	10015
		nBiomed	22-May-24	14d post 4 IN Vx	CD27$^+$B	46383#9	8383	1500

Supplementary Table S5. Germline genes, CDR3 sequences (CDR3-H and CDR3-L), binding activities to BA.1/BA.5 spike proteins, and epitopes of 45 mAbs

Name	V_gene_H	CDR3_H	V_gene_L	CDR3_L	BA.1 EC_{50} (nM)	BA.5 EC_{50} (nM)	Epitope
719-1	IGHV1-46	CARDRILPAAGWFDRW	IGLV3-21	CQVWDGSSDRYYVF	1.54	0.07	RBD
719-3	IGHV4-59	CARGGVPTTVFEGKGAGWFDPW	IGKV3-15	CQQYENWPGTF	0.22	1.76	NTD
719-4	IGHV5-51	CARLVVGDNFWSGYSDDAFDIW	IGLV1-47	CAAWDDTLNGVVF	0.61	2.04	NTD
719-5	IGHV3-53	CARDVLVRGVPYYFDYW	IGKV1-39	CQQSYSTPALTF	465.9	186.4	RBD
719-6	IGHV4-39	CARLSGYPDRFDYW	IGLV2-23	CCSYAGSSTYVF	1255	843.5	RBD
719-7	IGHV3-23	CAKVAARDGYNSDYW	IGKV3-11	CQQRSNWPPLTF	744	384.9	RBD
719-8	IGHV3-53	CARARKVAARHHNDAFDIW	IGKV3-15	CQQYNNWPPWTF	1039	560.6	RBD
719-9	IGHV7-81	CARENLGYNASGKYLRWFDPW	IGLV3-10	CHSTDSSNNPRRLF		no expression	
719-10	IGHV3-23	CAKGREFQLLQTRKFDWFDAW	IGKV4-1	CQQYYGTPLTF	/	/	/
719-11	IGHV3-33	CARDRLRGSGTTLDYW	IGKV3-15	CQQYYDPPPITF	/	/	/
719-12	IGHV1-18	CGRDRLRDFWSGGDYW	IGKV1D-33	CQQYDDLFSF	/	/	/
719-13	IGHV1-69	CARGRPFERPIDIW	IGKV3-20	CQQYGSSPITF	36.66	28.18	RBD
719-14	IGHV1-18	CARVYEAYCSRSSCYPNDYW	IGKV3-15	CQFDENWPPEYTF	0.08	0.05	RBD
719-15	IGHV4-4	CAREVGVVAGLYFDYW	IGLV3-21	CHVWDTSSDRVF	1119	667.4	RBD
719-16	IGHV4-39	CARVQLTGSGGRGHFGPW	IGKV4-1	CQQFYSSPLTL	732.1	630.3	RBD
719-17	IGHV4-38	CARGSYINSWSRSEFEYW	IGLV2-14	CSSYTSTTTRVF	732.1	630.3	RBD
719-18	IGHV3-23	CAKGRGSPNHYDHW	IGLV1-40	CQSYDNSLKAWVF	630.8	487.1	RBD
719-19	IGHV3-23	CAKDRELVAATRLFYFDSW	IGLV2-23	CCSYTGRSPYVF	0.07	0.04	NTD
719-20	IGHV3-23	CAKDRLTMLRGGMDVW	IGKV3-11	CQQRSNWPQTF	701.4	106.6	NTD
719-21	IGHV1-24	CGGDSVRYQFSLDSW	IGKV3-20	CQQYGRSPRIAF	22.68	8.80	NTD
719-22	IGHV4-34	CARSVSLPRGGVLNLW	IGKV3-15	CQQYHNWPQTF	1087	266	NTD
719-23	IGHV3-30	CARSGHSGGGRGPFAIW	IGKV1-39	CQQSYGTPYTF	32.87	15.73	RBD
719-24	IGHV3-30	CAKVSGGGRSLDYW	IGKV2-28	CMQALQIPWTF	49.41	18.94	NTD
719-25	IGHV3-23	CARGDRLQVQRLSLYSLDYW	IGLV3-19	CSSWDRSSNHPVF	30.40	14.05	RBD
719-27	IGHV4-39	CAGDKFSRIDASDYFDPW	IGLV3-9	CQVWATTTERVF	1547	243.1	NTD
719-28	IGHV1-46	CARSGAYYYGSGGRRASAGAEYFQH	IGKV1-8	CQQYYSYPPTF	15.99	7.13	RBD
719-29	IGHV1-18	CAREDQYGSGGRTGYYYGMDVW	IGKV1-8	CQQATSFPHTF	22.11	10.37	NTD
719-30	IGHV4-39	CARQWGSGGRGAWYFDYW	IGKV1-17	CLQHNSFPLTF		no expression	
719-31	IGHV3-43	CARDSGSGGRSYFDSW	IGKV3-20	CLHYGSSSYTF	/	/	/
719-32	IGHV3-73	CSRVPDDDFWSGGGRFDPW	IGLV6-57	CQSFDSTRLYVF	/	/	/
719-33	IGHV1-2	CARRKISGGGRALDTW	IGKV3-20	CQQYGSSLTF	/	/	/
719-34	IGHV2-5	CARLYYYDSSGGGRYDFW	IGLV3-1	CLAWDGSTAVF		no expression	
719-35	IGHV4-30	CARVVDMARATRIFDVW	IGLV3-21	CQVWDGNNAVVVF	/	/	/
719-36	IGHV3-7	CATLDVDMARLGALGVW	IGKV2-28	CMQSIDTRTF	34.27	17.90	RBD
719-37	IGHV2-5	CAHMRSHSVVVIAKTDKWFDPW	IGLV3-25	CQSGDSSGTVF	1.73	0.09	RBD
719-38	IGHV3-9	CAKDPRESSGYPRFDYW	IGLV6-57	CQSYDTDNPWVF	/	/	/
719-39	IGHV3-23	CARDRELTAATRLFYFDDW	IGLV2-23	CCSYATRSPYVF	0.06	0.04	NTD
719-40	IGHV3-33	CAREGSGTYYNPTISPDYW	IGKV2-30	CMQGIHWPWTF	4.11	0.29	RBD
719-41	IGHV3-7	CARTYYFDKTSPGLDQW	IGKV1-39	CHQSYGALTWTF	/	/	/

719-42	IGHV3-13	CARVVHVSPRRGNIYSYYMDMW	IGKV1-39	CQQTYSNPGSTF	0.93	0.32	RBD
719-43	IGHV4-59	CGRDLSELHGLYFHTDVW	IGKV3-11	CQQRHSWPSF	/	/	/
719-44	IGHV3-53	CARGVGEAASGTPGTRWFDPW	IGKV1-12	CQQSNGFPRTF	/	/	/
719-45	IGHV1-3	CARGNVGDSFDYW	IGKV4-1	CQQYYSLPLTF	/	/	/
719-46	IGHV4-4	CARDKARGIYYGSGSFSYYYGMDVW	IGKV3-20	CQQYGSSPLTF	19.09	13.65	RBD

The symbol "/" indicated a positive OD value at the highest concentration.

Supplementary Table S6. Demographics and vaccine regimens of eight donors used for cytokine and chemokine profiling in nasal washes.

Donor ID	Age (year)	Gender (F/M)	Doses of intramuscular vaccination with inactivated vaccine	Doses of intranasal vaccine	Interval between inactivated vaccine and intranasal vaccine (month)	Previous SARS-CoV-2 infection status
1	60	M	2	1	7	Uninfected
2	41	M	2	1	7	Uninfected
3	27	F	2	1	11	Uninfected
4	40	M	2	1	8	Uninfected
5	26	M	2	1	11	Uninfected
6	42	M	2	1	14	Uninfected
7	35	F	2	1	10	Uninfected
8	32	M	2	1	7	Uninfected

Supplementary Table S7. Yield of purified nasal sIgA, serum IgG, and serum IgA from six donors.

Donor ID	Nasal wash (mL)	Purified sIgA (μg)	Purified sIgA (μg/mL)	Serum (mL)	Purified IgA (mg)	Purified IgA (mg/mL)	Purified IgG (mg)	Purified IgG (mg/mL)
Donor 1	200	311.75	1.56	5.00	3.16	0.63	66.26	13.25
Donor 2	200	335.50	1.68	5.00	4.33	0.87	42.41	8.48
Donor 3	200	260.25	1.30	5.00	2.98	0.60	42.15	8.43
Donor 4	300	239.28	0.80	4.00	3.12	0.78	26.11	6.53
Donor 5	300	440.70	1.47	4.00	3.94	0.98	30.88	7.72
Donor 6	300	404.16	1.35	4.00	2.68	0.67	41.54	10.39
Mean±SD			1.36±0.31			0.75±0.15		9.13±2.38
