# Supplementary material for: Intranasal booster drives class switching and homing of memory B cells for mucosal IgA response
Source: JCI Insight. 2025 Dec 23;11(3):e198045. doi: 10.1172/jci.insight.198045 (PMC12892890; doi:10.1172/jci.insight.198045)

Full unedited gel for Fig.S1

Serum IgG

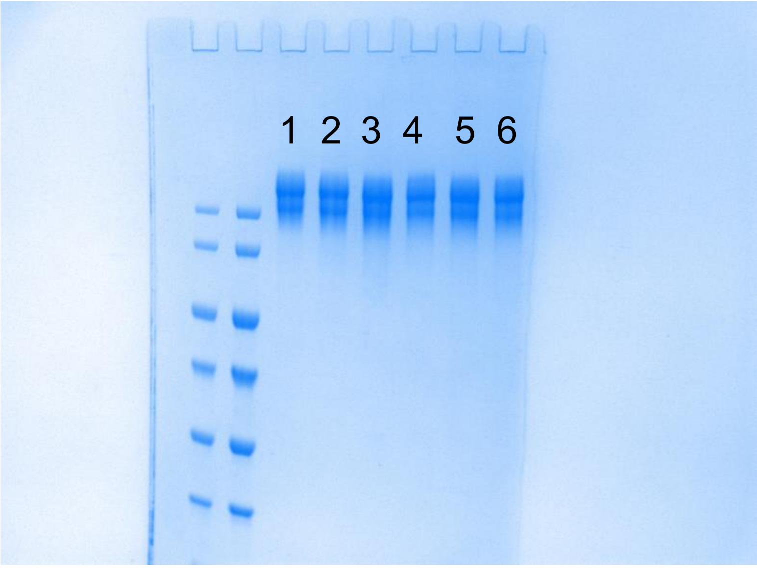

Nasal sIgA and serum IgA

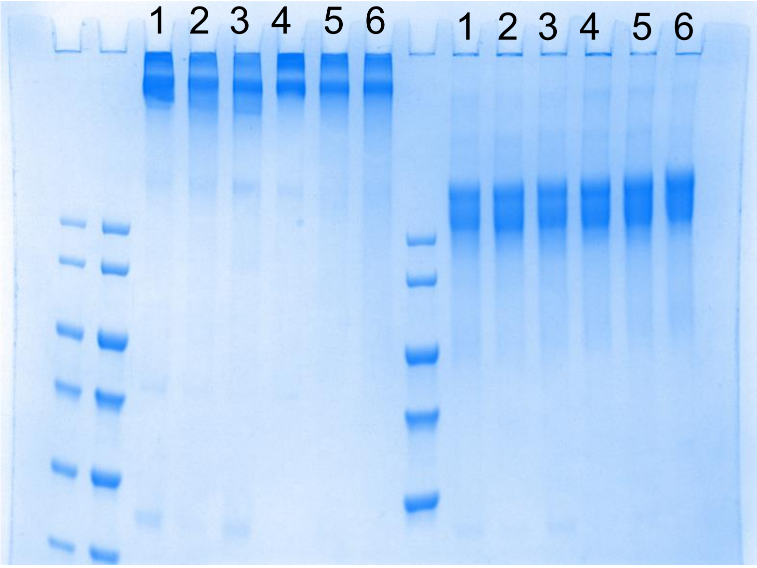

Serum IgG

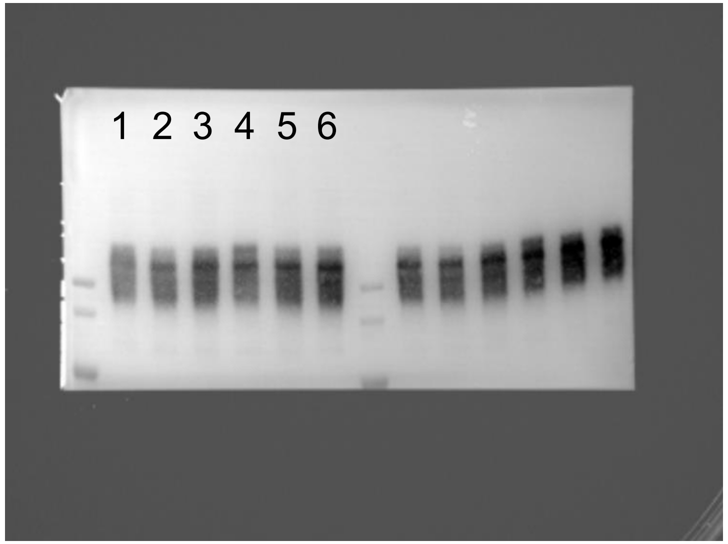

Nasal sIgA and serum IgA

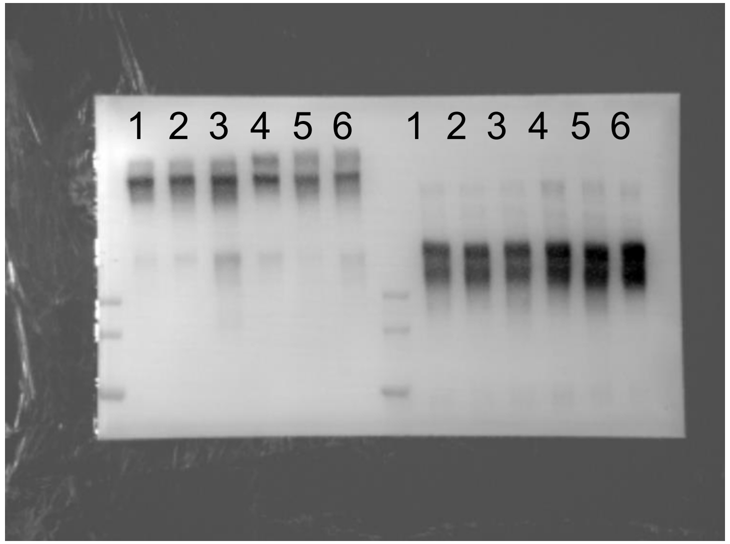

Full unedited gel for Fig.S2

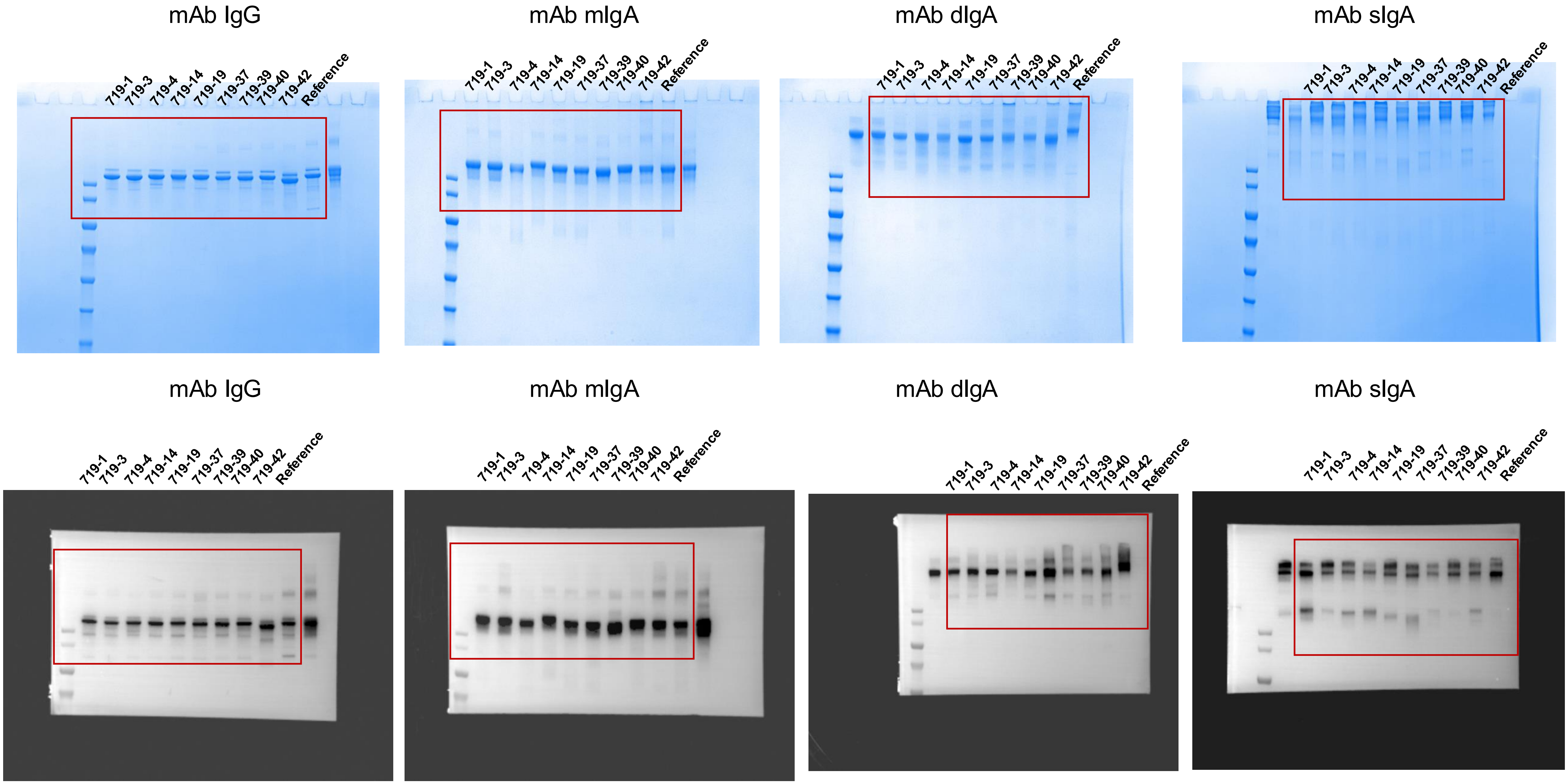

Supplement: Unedited blot and gel images [file jciinsight-11-198045-s160.pdf]
